# *Ruta graveolens* L. in Evidence‐Based Phytotherapy: Pharmacological Activities, Bioactive Compounds and Safety

**DOI:** 10.1002/ptr.70434

**Published:** 2026-09-01

**Authors:** Tetiana Serhiienko, Victoriya Georgiyants, Liudas Ivanauskas, Olha Mykhailenko

**Affiliations:** ^1^ Department of Pharmaceutical Chemistry National University of Pharmacy Kharkiv Ukraine; ^2^ Department of Analytical and Toxicological Chemistry Lithuanian University of Health Sciences Kaunas Lithuania; ^3^ Department of Pharmaceutical Biology Kiel University Kiel Germany; ^4^ Pharmacognosy and Phytotherapy UCL School of Pharmacy London UK

**Keywords:** biological activity, ethnopharmacology, phytochemistry, *Ruta graveolens*, toxicity

## Abstract

*Ruta graveolens* L. is widely used in traditional European, Asian, African, and South American medicine for digestive, nervous, cardiovascular, respiratory, and reproductive disorders. Despite its long‐standing ethnomedical relevance, the translation into evidence‐based phytotherapy remains limited due to fragmented pharmacological data and well‐documented safety concerns. This review critically synthesizes ethnomedical use, phytochemical composition, pharmacological activities, and toxicological risks of *R. graveolens* , aiming to evaluate its therapeutic potential and identify key research gaps. A systematic search and critical analysis were conducted following PRISMA guidelines using PubMed, ScienceDirect, and Google Scholar. Ethnomedicinal data were reviewed without time limits, while phytochemical and pharmacological studies published between 2014 and 2025 were analysed in detail. More than 160 compounds (including furanocoumarins, alkaloids, flavonoids, and others) have been identified by HPLC, CC, GC–MS, and NMR methods, of which 38 are characteristic markers. The plant exhibits a wide spectrum of activity, from antimicrobial to antitumor. However, due to the content of furanocoumarins and alkaloids, the extracts have phototoxic, abortifacient, hepatotoxic, and nephrotoxic effects, which highlights safety concerns in the absence of dosage control. Although *R. graveolens* represents a chemically diverse source of bioactive compounds with promising pharmacological potential, its integration into modern phytotherapy requires rigorous toxicological evaluation, extract standardization, and well‐designed preclinical and clinical studies. Future research should prioritize development of standardized extracts with defined phytochemical profiles, comprehensive dose‐dependent toxicological assessment, and translational studies bridging experimental findings with clinical applications with particular attention to safe therapeutic windows and less toxic bioactive constituents.

## Graphical abstract

Graphical representation of ethnomedical uses, major bioactive compounds, pharmacological potential and toxicological risks of Ruta graveolens L.

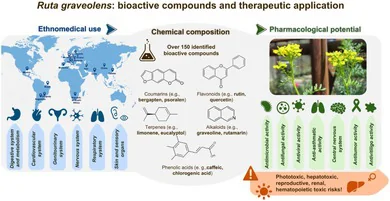

**Abbreviations**
A1: neuronal cells (differentiated/undifferentiated)
A375: human melanoma cell line
AchE: acetylcholinesterase
AKT: protein kinase B
ALT: alanine aminotransferase
AMPA: α‐amino‐3‐hydroxy‐5‐methyl‐4‐isoxazolepropionic acid
AST: aspartate aminotransferase
Atg5, Atg7: autophagy‐related genes/proteins 5 and 7
B16‐F1/B16F10: mouse melanoma cell lines
BAX: Bcl‐2‐associated X protein
BChE (BuChE): butyrylcholinesterase
Bcl‐2: B‐cell lymphoma 2
BDNF: brain derived neurotrophic factor
Beclin‐1: autophagy‐related protein beclin‐1
C3: complement component 3
CC: column chromatography
CC_50_
: half‐maximal cytotoxic concentration
C‐glycoside: carbon‐linked glycoside
CH: centesimal hahnemannian dilution
CLint: intrinsic clearance
cm: centimeter/centimetre
CREB: cAMP response element‐binding protein
DAD: diode array detector
D‐GalN/LPS: D‐galactosamine/lipopolysaccharide
DMBA: 7,12‐dimethylbenz(a)anthracene‐induced skin cancer
DNA: deoxyribonucleic acid
E.Z.: estimated binding energy
EBV: Epstein–Barr virus
EC_50_
: half maximal effective concentration
EGFR: epidermal growth factor receptor
EIMS: electron ionization mass spectrometry
ERK1/2: extracellular signal‐regulated kinase 1/2
ESI: electrospray ionization
FC: flash chromatography on silica gel
FEV1: forced expiratory volume in one second
FVC: forced vital capacity
GC: gas chromatography
GC–MS: gas chromatography–mass spectrometry
GC‐qTOF‐MS: gas chromatography–quadrupole time‐of‐flight mass spectrometry
GFAP: glial fibrillary acidic protein
GSCs: glioma Stem Cells
HaCaT: immortalized human keratinocyte cell line
HEK‐293: human embryonic kidney cells
HeLa: human cervical cancer cell line
HepG2: human liver cancer cell line
HK‐2: human kidney proximal tubular epithelial cells
hMAO‐A/hMAO‐B: human monoamine oxidase A/B
HPLC: high‐performance liquid chromatography
HPLC‐DAD: high‐performance liquid chromatography with diode array detection
HPLC‐MS: high‐performance liquid chromatography–mass spectrometry
HR‐ESI‐MS: high‐resolution electrospray ionization mass spectrometry
HT‐29: human colon cancer cell line
IC_50_
: half maximal inhibitory concentration
IFN‐γ: interferon gamma
IgA: immunoglobulin A
Ig‐E: immunoglobulin E
IL‐10: interleukin 10
IL‐1β: interleukin 1 beta
IL‐4: interleukin 4
IL‐6: interleukin 6
IL‐7: interleukin 7
InhA: enoyl‐acyl carrier protein reductase
iNOS: inducible nitric oxide synthase
JAK1/STAT3: Janus kinase 1/signal transducer and activator of transcription 3
Jurkat: human T lymphocyte cell line (leukemia)
Kd: dissociation constant
KEGG: kyoto encyclopedia of genes and genomes
Ki: inhibition constant
LC3‐II: microtubule‐associated protein 1A/1B‐light chain 3‐II
LPS: lipopolysaccharide
m: meter/metre
MBC: minimum bactericidal concentration
MCF‐7: human breast cancer cell line
MCO: 8‐methoxychromen‐2‐one
MDA: malondialdehyde
MFC: minimum fungicidal concentration
MIC: minimum inhibitory concentration
MITF: microphthalmia‐associated transcription factor
mRNA: messenger ribonucleic acid
MRSA: methicillin‐resistant staphylococcus aureus
MT‐1, MT‐2: human adult T‐cell leukemia/lymphoma cell lines
mTOR: mammalian target of rapamycin
NF‐κB: nuclear factor kappa‐light‐chain‐enhancer of activated B cells
NO: nitric oxide
NRK‐49F: normal rat kidney fibroblasts
NSCLC: non‐small‐cell lung cancer
O‐glucoside: oxygen‐linked glucoside
P53: tumor protein p53
pAkt: phosphorylated Akt
PARP: poly (ADP‐ribose) polymerase
PBMC: peripheral blood mononuclear cells
PI3K/AKT: phosphoinositide 3‐kinase (PI3K)/protein kinase B (Akt) pathway
PIG3V: human melanocytes (used in vitiligo models)
p‐TLC: preparative thin layer chromatography
qTOF: quadrupole time‐of‐flight
ROS: reactive oxygen species
SARS‐CoV‐2: severe acute respiratory syndrome coronavirus 2
SI: selectivity index
SMAD: mothers against decapentaplegic homolog
SOD: superoxide dismutase
S‐protein: spike protein
t_1/2_
: half‐life
TAS2R: taste receptor type 2 (bitter taste receptor)
TGF‐β: transforming growth factor beta
tMCAO: transient middle cerebral artery occlusion
TMEM119: transmembrane protein 119
TNF‐α: tumor necrosis factor alpha
TRP: transient receptor potential
TRPA1: transient receptor potential ankyrin 1
TRPM5: transient receptor potential melastatin 5
TRPM8: transient receptor potential melastatin 8
TRPV1: transient receptor potential vanilloid 1
U251MG: human glioblastoma cell line
U87MG, U138, C6: glioblastoma cell lines
UPLC: ultra‐performance liquid chromatography
UPLC‐ESI‐MS/MS: ultra‐performance liquid chromatography–electrospray ionization–tandem mass spectrometry
UPLC‐QTOF‐MS: ultra‐performance liquid chromatography–quadrupole time‐of‐flight mass spectrometry
ZOI: zone of inhibition
α‐SMA: alpha‐smooth muscle actin

## 1 Introduction

Phytotherapy is a traditional medical practice in various cultures. Natural remedies have been actively used since ancient times, even before the advent of well‐known synthetic drugs and pharmaceutical companies (Ravindran et al. 2012). Currently, there is a new trend toward the revival of herbal therapy in the context of evidence‐based medicine. This is due to the need to expand therapeutic options alongside modern medicines. One of the traditional medicinal plants is *R. graveolens* , commonly known as rue. It is a perennial herbaceous plant originating from the Mediterranean and cultivated in various regions of the world (Fuselier et al. 2022). It is distinguished by its characteristic aroma and bitter taste, which has long determined its use in local medicine, rituals, and even cooking (Ravindran et al. 2012). Historically, *R. graveolens* has occupied a prominent place in ethnomedicine: references to it can be found in the works of ancient Greek and Roman physicians, medieval European herbals, and in the local traditions of Asia, Latin America, and Eastern Europe (Pollio et al. 2008). In many cultures, the plant was attributed with protective, healing, and symbolic properties—from use against infections and inflammation to use in spiritual and ritual practices (Colucci‐D'Amato and Cimaglia 2020). Today, it continues to be used in local remedies, particularly in the Balkans, Ukraine, and South America (Łuczaj et al. 2021; Kozlowska et al. 2018).

Phytochemical studies have shown that *R. graveolens* is a rich source of bioactive compounds, including alkaloids, flavonoids, coumarins, and essential oils (Nahar et al. 2021; Fuselier et al. 2022; Ulubelen and Öztürk 2006). Characteristic metabolites such as rutin, quercetin derivatives, and furanocoumarins (bergapten, xanthotoxin) are of interest due to their potential pharmacological significance (Asgharian et al. 2020; Águila et al. 2015; Jash et al. 2024; Fuselier et al. 2022). These compounds exhibit a wide range of biological activities, including antimicrobial, antioxidant, anti‐inflammatory, and neurotropic effects, which are also characteristic of *R. graveolens* extracts (Forouhar et al. 2019). At the same time, *R. graveolens* is characterized by pronounced toxicological properties. Phototoxicity associated with furanocoumarins, reproductive toxicity, and other risks limit its therapeutic use and emphasize the importance of careful analysis of the benefit/risk ratio (Abebe 2021; Seak and Lin 2007). These facts indicate the need for further study of the plant for its rational use in medicine.

While *R. graveolens* is extensively documented in scientific literature and is well‐known for its ethnomedical significance, its integration into modern clinical practice remains complex due to the variability of its pharmacological effects and the need for rigorous standardization. Despite the existence of individual publications on the chemical composition, pharmacology, or traditional use (Mokhtar et al. 2022; Reyes‐Vaquero et al. 2021; Szewczyk et al. 2022) and several recent comprehensive reviews (Ali et al. 2024; Shahrajabian 2023; Dwivedi and Verma 2024), there is still a need for a more structured and updated synthesis. While the aforementioned studies provide a broad overview, they often lack a systematic methodology for source selection and critical evaluation. By applying a systematic approach to identify specific gaps in current clinical evidence, this review aims to integrate existing data on the historical ethnobotanical significance, botanical characteristics, phytochemical profile, pharmacological effects, and safety of *R. graveolens* and provide a definitive roadmap for the safe and rational integration into modern evidence‐based phytotherapeutic practice. Summarizing the available knowledge will allow us to identify key areas for further research and lay the groundwork for more in‐depth study.

## 2 Materials and Methodology

The review followed the PRISMA‐ScR (Preferred Reporting Items for Systematic Reviews and Meta‐Analyses Extension for Scoping Reviews) guidelines (Page et al. 2021). The PRISMA flow diagram illustrating the study selection process is presented in Figure 1.

**FIGURE 1:**
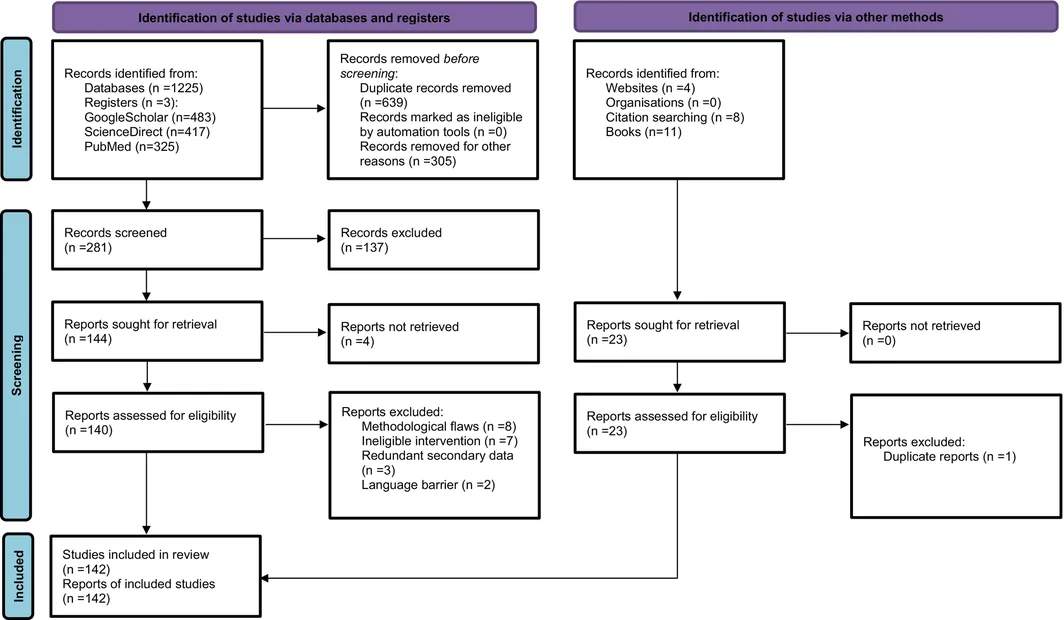
PRISMA flow diagram summarizes the search strategy and selection process and inclusion criteria. This diagram illustrates the systematic search strategy conducted across PubMed, ScienceDirect, and Google Scholar, summarizing the initial identification of 1225 records and the final selection of 142 studies based on strict eligibility criteria.

A systematic search and critical analysis of existing studies on *Ruta graveolens* was conducted. An electronic search was performed in the PubMed, ScienceDirect, and Google Scholar databases. The following keywords were used: “ *Ruta graveolens* AND phytochemistry”; “ *Ruta graveolens* AND ethnomedicine OR traditional use OR traditional medicine”; “ *Ruta graveolens* AND essential oil OR volatile compounds”; “ *Ruta graveolens* AND alkaloids OR flavonoids OR coumarins OR terpenes OR phenolic compounds”; “ *Ruta graveolens* AND pharmacological activity OR antimicrobial OR antifungal OR neuroprotective OR anticancer”. Where applicable, Medical Subject Headings and database‐specific indexing terms were applied to increase search sensitivity. In addition, the reference lists of relevant publications were manually screened to identify potentially eligible studies not retrieved through database searching.

Inclusion criteria: Books and book chapters, original articles, clinical and preclinical studies, as well as peer‐reviewed reviews directly related to *R. graveolens* were included in the analysis. Only sources indexed in international databases were considered. Preference was given to studies presenting experimental data, including phytochemical investigations, isolation and purification of compounds using chromatographic techniques, structural identification employing spectroscopic and analytical methods, and evaluation of biological activity in in vitro, in vivo, or in silico models.

Publications in English, Ukrainian, and Russian were included in the review (for Russian‐language publications, only those published before 2013). The research team, being based in Ukraine, possesses native and high‐level proficiency in Ukrainian and Russian, therefore, relevant studies in these languages were analyzed in their original form without the need for external translation services. Key data and findings from these non‐English sources were translated into English by the authors during the data synthesis and manuscript preparation stages. No time restrictions were set for ethnomedical data, as it was important to take historical sources into account. For studies on chemical composition and pharmacological activity, the time frame was 2014–2026, which allowed us to focus on the most recent data.

Exclusion criteria: Publications that did not meet the review objectives were excluded, as were conference abstracts and materials, editorial articles, comments, blogs, and other non‐scientific sources due to their limited scientific value. Duplicate records were removed.

Selection of publications: Relevance was assessed based on titles, abstracts, and full texts. All documents were reviewed independently by several researchers. In case of disagreement regarding the inclusion of a publication, consensus was reached through discussion. Works directly related to *R. graveolens* were selected for the final analysis.

Structural formulas of the identified compounds were prepared using BIOVIA Draw 2017 R2.

## 3 Geographical Distribution

*Ruta graveolens* L. (genus Ruta, family Rutaceae) is a widely recognized and well‐documented species with a long history of human interaction, originating from southern Europe and the Mediterranean (Salvo et al. 2010). Its natural habitat ranges from the Crimean Peninsula to the Balkans (Pollio et al. 2008). It is also believed that its native countries are Yugoslavia, Albania, Turkey, Bulgaria, and Ukraine (Figure 2) (GRIN‐Global 2025a; Plants of the World Online 2025).

**FIGURE 2:**
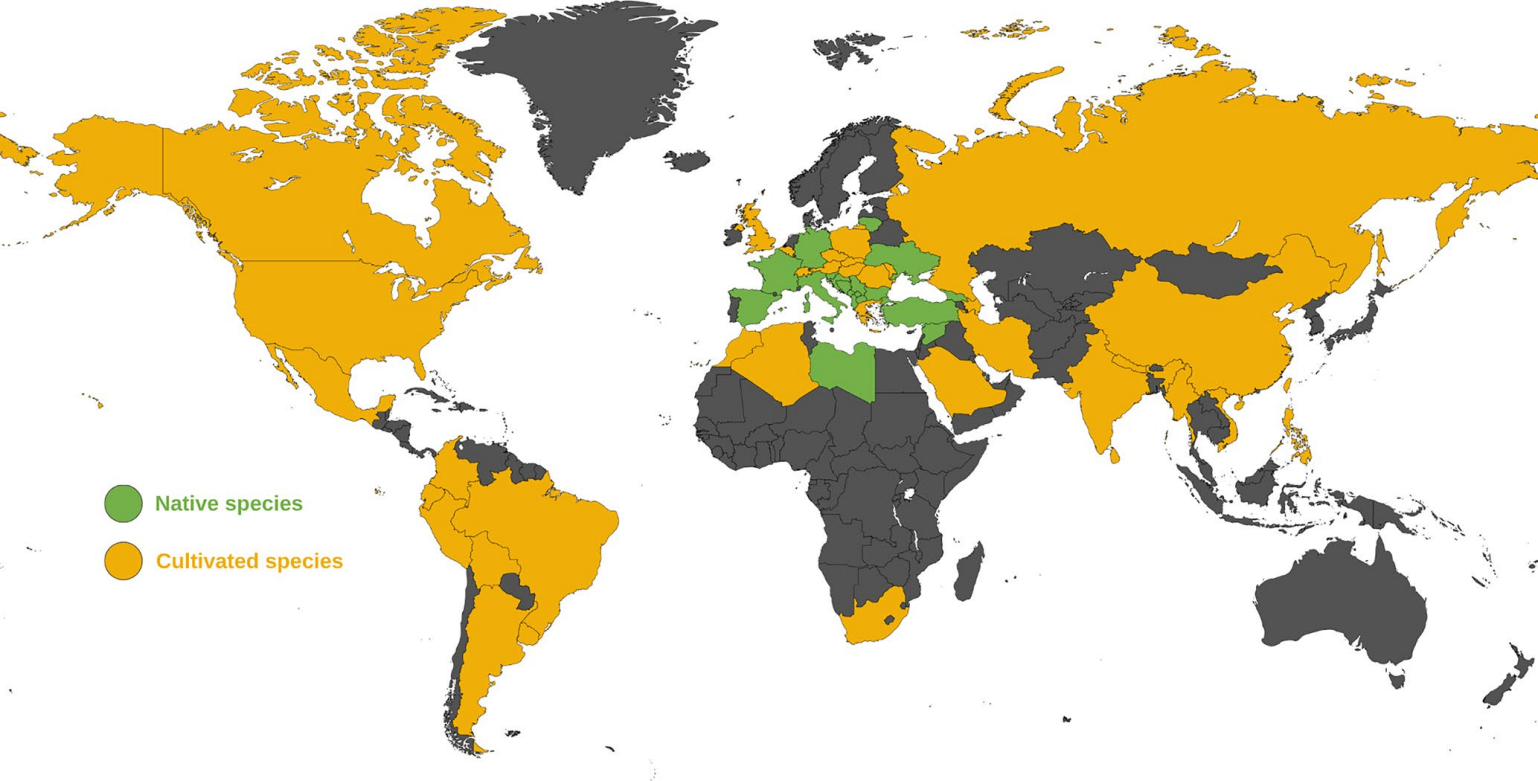
Global distribution of *R. graveolens* showing native and cultivated regions. The actual species is shown in green. Yellow indicates the country where *R. graeolens* was introduced and where it is currently cultivated for various purposes. [Correction added on 2 November, after first online publication: Figure 2 has been corrected.]

In the wild, it grows on dry rocky mountain slopes and river banks in the grassy cover of low‐growing evergreen shrubs. The plant prefers the temperate climate characteristic of its native range. It is often found at altitudes of up to 1200 m above sea level, where it occupies open and well‐lit areas. Over time, due to the active development of migration and international trade, *R. graveolens* has been widely introduced and is now successfully cultivated in regions with similar climatic conditions in approximately 40 countries in Asia, Europe, North and South America, and Africa. Its widespread distribution is also due to the plant's adaptive properties; integration into new ecosystems does not usually threaten overall biodiversity and has no negative consequences (Khareba et al. 2016).

For effective cultivation, the plant needs sufficient light and moisture, protection from the wind, and soil rich in fertilizers and minerals. However, it is believed that it can adapt well to poor and infertile soils. It is usually cultivated in one place for 5–8 years (Khareba et al. 2016).

Thus, *R. graveolens* has become widespread both in the wild and in cultivation due to its adaptability and resistance to various climatic conditions. This makes it a promising subject for further study in various fields.

## 4 Botanical Description

*R. graveolens* is a perennial herbaceous plant or small shrub, 20–70 cm tall (Figure 3). The stems are erect, branched, glabrous, bluish‐green in color, sometimes woody at the base. The leaves are light green with a bluish tinge, alternately arranged, ovate, dissected, with an entire or finely serrated margin and numerous essential oil glands on the surface, which cause a characteristic odor that resembles the aroma of tomatoes when dried. The flowers are bright yellow, small, and arranged in a brush‐like inflorescence. The fruit is a four‐celled dry hard capsule. The seeds are small and brown in color. The plant is poisonous. In studies for the preparation of medicinal raw materials, the above‐ground parts of the plant are usually used, namely the stems, leaves, flowers, and buds (Khareba et al. 2016; Korniievskyi et al. 2017; Lebeda et al. 1992). Microscopic examination of the Rutaceae family has identified characteristics that distinguish *R. graveolens* from other plants. This is important because some species are very similar, and there have been cases where they have been confused in experiments due to their similar morphological characteristics. Unique characteristics include: a soft and pleasant aroma of raw materials; wavy or serrated petals; a blunt tip of the fruit segments; no trichomes, either glandular or non‐glandular; wide fiber lumen; abundant calcium rosettes in the leaves; rare calcium rosettes in the stem. These characteristics will help to identify the plant species correctly, which is an initial and important step in any research for further work (Kannan and Babu 2012).

**FIGURE 3:**
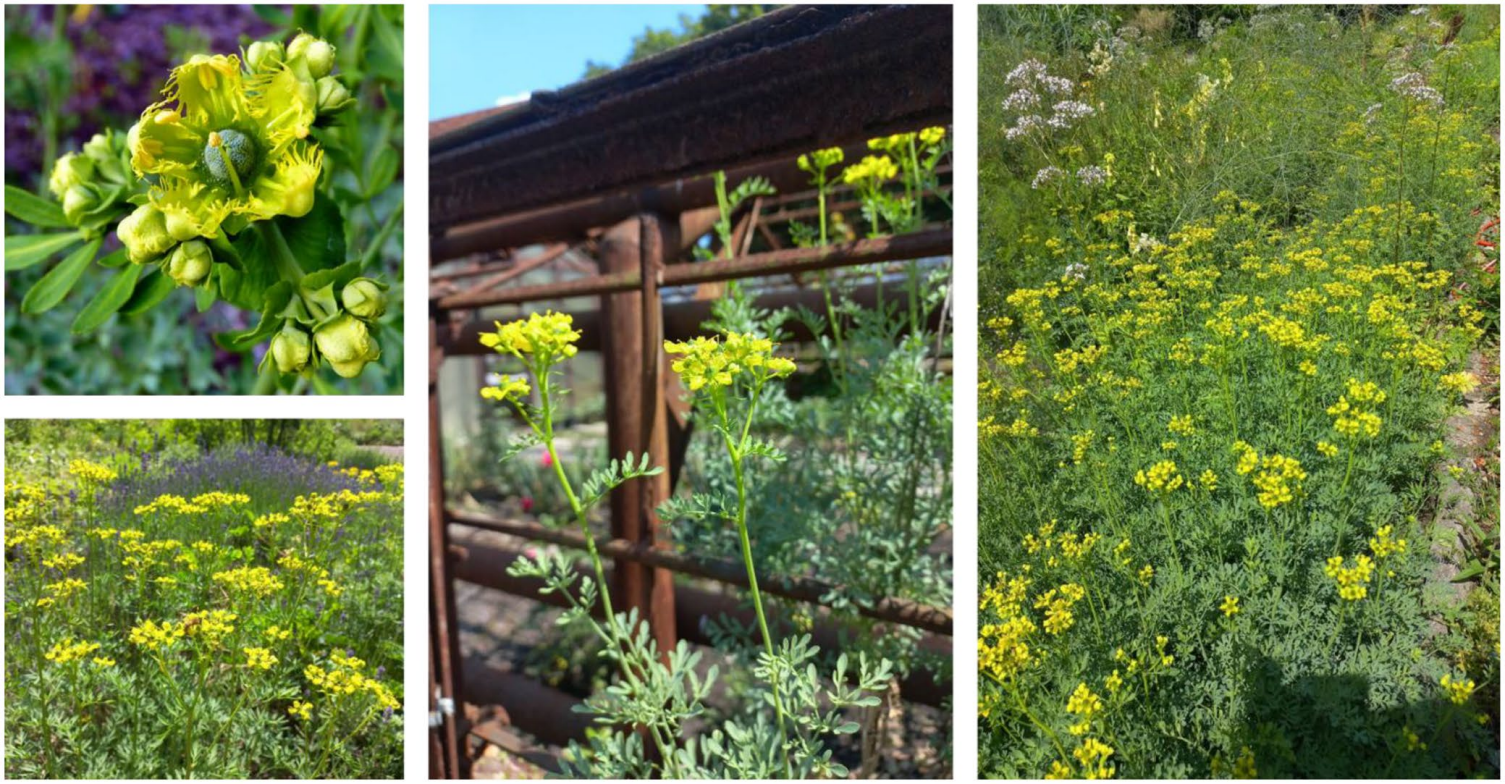
Appearance of *R. graveolens* L. (period of mass flowering), photo taken at the Botanical Garden of Ivan Franko National University of Lviv, July 2023, photo by Tetiana Serhiienko. The photograph depicts the plant in its mass flowering phase; the yellow tetramerous flowers and pinnately compound leaves are highlighted as primary markers for raw material identification.

## 5 Ethnomedical Use

Throughout human history, medicinal plants have been an important means of therapy and health maintenance, and each culture has developed its own set of ethnomedical knowledge, determined by natural resources, climate, and traditions. *R. graveolens* occupies a special place in the traditional medicine of many peoples, from the Mediterranean to South America. The breadth of its application is reflected in ancient and medieval texts, folk tales, songs, and botanical literature. This centuries‐old experience provides the basis for modern research and the search for new pharmacological applications in evidence‐based medicine. According to some sources, the name of the plant comes from the Greek word “rua”, which means “to release”, which probably reflects its traditional role as a remedy for various diseases. *R. graveolens* is known by several common names in different languages. In English, it is usually called “common rue” or “rue”. In French, the plant is known as “rue fétideє” or “rue officinale”, in German—“Weinraute”, portugal—“arruda”, in Spanish—“ruda” or “ruda de monte”, in Dutch—“ruit”. In Swedish, it is called “vinruta”, while in Ukrainian it is transliterated as “ruta”. In Korean, the local name is transcribed as “unhyang” (GRIN‐Global 2025b). Most Western European languages have similar names for rue, derived from the Latin “ruta”, which was borrowed from the Greek “rhyte” [ῥυτή]. The ultimate origin of this word remains unknown. Interestingly, the names for rue in some languages have homonyms. The Latin species name “graveolens”, which rue shares with a number of other aromatic plants, means “strong‐smelling”: “gravis”—“heavy”, “olens”—present participle of “olere”—“to smell” (Allred 2024).

In the works of ancient authors, *R. graveolens* is described as a broad‐spectrum remedy. Thus, in ancient times, Hippocrates mentions the plant in his work “Materia Medica” (D’Andrea 1982). He attributed diuretic properties to plant (Tobyn et al. 2011). Later, Dioscorides described rue in his work “De Materia Medica,” one of the most valuable botanical treatises of antiquity (D’Andrea 1982). He listed more than a dozen external applications for joints, abdomen, head, eyes, and ears to relieve pain, ground with honey for difficult breathing of the uterus, in cases of intestinal inflammation, vitiligo, leukemia, lichen‐like rashes, erysipelas, poor vision, or dandruff (Tobyn et al. 2011). Plinius Maior mentioned rue in his work “Naturalis Historia,” describing recipes for conditions such as psoriasis, warts, colic, nosebleeds, and a number of others (D’Andrea 1982). He warned about the risks of overdose with the plant juice (Tobyn et al. 2011). Apuleius prescribed its use for pain, inflammation, ears, eyes, kidneys, skin. Galen highlighted its use for shoulder diseases and to regulate menstruation, notably, according to Galenic theory, *R. graveolens* was utilized to “dry up” or congeal the “seed” (semen), acting as an anaphrodisiac to suppress sexual desire and as a contraceptive agent to prevent conception (Tobyn et al. 2011). The ancient Greek gynecologist Soranus, in turn, mentioned the vaginal use of rue, which he interpreted as abortive (Tobyn et al. 2011). Dioscorides, Plinius Maior (D’Andrea 1982) and Oribasius also recommended the use of rue, as already mentioned, and Quintus Serenus indicated that a mixture of “eggs, rue, and dill” promotes abortion (Gamal 2023). The herb was even mentioned in the Arab world in major syntheses since the Middle Ages. Thus, Al‐Biruni wrote about the raw material in his “Book of Medicines” (Kitab al‐Saydalah), Al‐Ghafiki in his work “Book of Simple Medicines” (Kitab al‐Adwiyah al‐Mufradah), Ibn Al‐Baytar in his work “Book of Medicine and Food” (Kitab al‐Jami' li‐Mufradat al‐Adwiya wa‐l‐Aghdhiya) (Tobyn et al. 2011). The work “Taqwim al‐Sihha” (Taqwim al‐Sihha) by Ibn Butlan also contains references to raw materials (Pollio et al. 2008). Ibn Ridwan wrote about rue in his medical work “Treatise on the Preservation of Health” (Gamal 2023). Ibn Sina prescribed the herb for the treatment of baldness, to reduce libido, and for paralysis (Tobyn et al. 2011). According to legend, King Mithridates VI Eupator of Asia Minor used rue as an antidote, which allegedly allowed him to survive numerous attempts at poisoning (D’Andrea 1982). There is little evidence of references to rue by authors such as Serapio, Hildegard of Bingen, Macer, Gerard, Culpeper, Parkinson, and Bauhin (Tobyn et al. 2011). Gerard's herbal tells us that it stimulates urination, reduces nausea, expels dead babies and afterbirth, relieves ear pain, combats poisoning and ulcers, stops nosebleeds, and counteracts the plague. Calpeper described the plant as the “Grass of Grace,” which induces urination and female discharge, acts as an antidote, reduces pain in the chest, joints, and gout, and treats worms, coughs, pneumonia, eye diseases, and more. In European medieval traditions, rue was also widely used to protect against poison, snake and scorpion bites, plague infections, and as an anti‐epidemic agent (Plinius Secundus 2018). Over time, the use of rue evolved, given its possible toxicity and abortive effect. Thus, the plant remains valuable in the treatment of various diseases, especially in places where it grows naturally. Subsequently, traditional medicine switched to safer external and internal use as an anthelmintic, antirheumatic agent, against lung diseases, and others (Pollio et al. 2008).

*R. graveolens* is present in the traditional medicine of many cultures due to its medicinal properties and is used in various forms, the most common of which are infusions, decoctions, alcohol tinctures, powders, fresh plant parts, powders, and essential oils (Table 1).

**TABLE 1:** Traditional uses of *R. graveolens* in different cultures.

	Ukraine	Lithuania	Germany	Italy	Poland	Romania	Bosnia and Herzegovina	India	Taiwan	China	North Africa	Uruguay	Morocco	Iran	South America	Mexico	Brazil	Argentina
A. Digestive system and metabolism																		
Stomach pain/Stomach cramps/Colic				+						+	+		+			+	+	+
Intestinal pain																	+	
Flatulence	+			+				+										
Loss of appetite/Digestive disorders/Improved appetite/Indigestion/Discomfort/Vomiting	+		+	+	+						+		+		+		+	+
Colitis								+										
Diarrhea/Dysentery						+				+						+	+	
Diabetes											+		+					
Aphthous stomatitis						+				+								
C. Cardiovascular system																		
Heart Disease/Heart Problems							+		+	+	+							
Tachycardia/Heartbeat Normalization				+					+									
Hypertension													+					
Thrombosis																	+	
G. Genitourinary system and sex hormones																		
Menstrual pain/Cramps	+		+	+									+				+	
Stimulation of menstruation (amenorrhea)	+	+		+				+		+	+			+	+	+	+	
Inflammation/Diseases of the ovaries, uterus												+					+	
Ease of childbirth											+							
Abortive agent	+	+		+				+		+		+	+	+		+	+	
Contraception (for men)	+													+				
Kidney diseases (so‐called gout)					+			+			+		+				+	
Bladder diseases											+							
Cholelithiasis	+																	
Liver diseases													+					
J. Antimicrobials																		
Fever/Flu, as an anti‐inflammatory, diaphoretic, anti‐infective (fever, tetanus, cholera)			+	+	+	+		+		+	+	+	+	+		+	+	
Poisonous snake/Insect bites, as an antidot		+		+	+					+		+	+			+		
M. Musculoskeletal system																		
Muscle pain/Spasms/Fibromyalgia	+	+	+				+	+		+		+		+	+	+	+	
Spasms										+					+			
Rheumatism	+									+			+				+	
Arthrosis/Arthritis								+				+					+	
Sciatica								+										
Rickets	+																	
Bruises/Bruises/Wounds	+			+												+	+	+
N. Nervous system																		
Dizziness				+								+				+		
Multiple sclerosis	+																	
Seizures/Epilepsy					+			+		+	+	+	+					
Headache/Toothache/Paralysis/Neuralgia/Tinnitus								+		+	+		+	+		+	+	
Nervousness/Anxiety, as a sedative	+		+					+					+	+		+	+	
Hysteria	+			+							+	+						
Postpartum depression																	+	
R. Respiratory system																		
Bronchitis/Cough, as an expectorant	+			+	+	+		+			+		+			+	+	
S. Skin and sensory organs																		
Dermatitis/Eczema/Ulcers/Boils/Vitiligo	+							+		+		+	+				+	
Eye Diseases (conjunctivitis, inflammation, improved vision)	+							+					+				+	+
Ear Diseases	+										+					+	+	
P. Antiparasitics, insecticides and repellents																		
Against lice, fleas, mosquitoes, as a repellent			+	+							+	+					+	+
Against worms				+							+		+	+	+		+	

### 5.1 Argentina

In traditional Argentine medicine, *R. graveolens* was used in a similar way to the previous countries, namely, a decoction of the leaves was used to wash the eyes in case of diseases, and compresses soaked in a decoction of the above‐ground parts of the plant were applied externally to wounds and injuries (Martínez and Luján 2011).

### 5.2 Bosnia and Herzegovina

In traditional medicine in Bosnia and Herzegovina, the plant was mainly used externally. Essential oil was used to rub the back for pain relief. The raw material was also used internally for heart disease, although specific dosage forms are rarely described in the sources; infusions or decoctions were probably used (Ginko et al. 2022).

### 5.3 Canada

In traditional Canadian medicine, *R. graveolens* herb was used to treat animals for tapeworms and roundworms (Lans et al. 2007).

### 5.4 China

In traditional Chinese medicine, *R. graveolens* has been used as a dried or fresh whole plant, including roots, stems, and leaves, mainly in the form of decoctions, infusions, or topical preparations. It has been prescribed to clear heat and toxins, cool blood, disperse blood stasis, and relieve pain. Indications also include colds and fever, rheumatism, paralysis, convulsions, colic, diarrhea, mouth ulcers, insect and snake bites, amenorrhea and abortion, eczema, and amenorrhea (Luo et al. 2024). In addition, the plant has long been used as a local remedy in China for the treatment of vitiligo and psoriasis (Ainiwaer et al. 2022; Ainiwaer et al. 2023).

### 5.5 Germany

In traditional German medicine, *R. graveolens* was used mainly for its antispasmodic, anti‐inflammatory, and sedative properties. It was used externally to wash the head against lice—this method of use was considered effective due to the possible repellent properties of the essential oils contained in the raw material (Medicinal Herb 2025).

### 5.6 Lithuania

In traditional Lithuanian medicine, *R. graveolens* has been known since ancient times and was used as a medicinal plant to stimulate menstruation, induce abortions, relieve spasms, and as a remedy for snake bites (Tumėnas 2023).

### 5.7 India

In traditional Indian medicine, *R. graveolens* was used in Ayurveda, Unani, and Siddha systems in various dosage forms—powders, infusions, juice, decoctions, tinctures, and topical ointments. The powder was used to stimulate menstruation and as an abortifacient. Mixtures with *R. graveolens* juice or extract were used topically, often in combination with honey, for nervous disorders, paralysis, and tremors. A decoction of the plant in the form of enemas was prescribed to relieve flatulence and symptoms of colitis. The juice was used as an expectorant for bronchitis and respiratory diseases. The tincture was used to improve vision and relieve eye fatigue and strain. In addition, a fairly wide range of indications is described, both in internal and external dosage forms, due to its therapeutic effect (Khare 2007; Parray et al. 2012).

### 5.8 Iran

According to Avicenna's medical book “The Canon of Medicine,” *R. graveolens* was used in traditional Iranian medicine as an antispasmodic, anthelmintic, analgesic, anti‐inflammatory, antifungal, and sedative (Sina 2005). It was widely used as an abortifacient–emmenagogue (Madari and Jacobs 2004). Additionally, the plant served as contraception for men (Harat et al. 2008).

### 5.9 Italy

In traditional Italian medicine, *R. graveolens* was used in various forms, most often in the form of dried leaves, powder, infusions, decoctions, distilled oil, and fresh juice. For example, dried leaves collected during flowering, with the addition of boiling water, were used as an antipyretic agent that stimulated sweating. It was widely known for its powerful antispasmodic and choleretic effect and its ability to induce menstrual bleeding. The raw material was recommended against worms and snake bites (Riddle 1992). Since ancient times, *R. graveolens* has been considered a remedy for infections, inflammation, and even the plague. Fresh leaves were applied topically to relieve headaches, improve blood circulation, and treat bruises. Compresses soaked in the plant's decoction were applied to the chest for bronchitis, and the squeezed juice helped with nervous disorders. It was recommended to chew a few leaves of the fresh plant in case of dizziness, hysterical spasms, increased heartbeat, and other conditions (Grieve 1971). In addition, *R. graveolens* was used as a repellent, fungicide, and phytotoxic agent in agriculture. Extracts of the herb were used as an antidote for some snakes and scorpions (Mancuso et al. 2015). The plant was also actively used externally, placed under the bed to get rid of bedbugs, rubbed on the scalp with a decoction for lice infestation, or hung crushed leaves around the neck as a remedy for worms (Guarrera 1999).

### 5.10 Mexico and the Southwestern United States

There are quite a few references to the use of *R. graveolens* in traditional medicine in Mexico. An infusion of the herb was taken internally for a wide range of indications, including digestive problems, nervous system problems, inflammation, and various types of pain (Josabad Alonso‐Castro et al. 2012). Information on dosage varies. There have been cases of poisoning from internal consumption of large amounts of the raw material; for example, the use of about 100 g of leaves in a single dose has been documented for emmenagogue purposes, but it is noted that such high concentrations are considered dangerous and potentially fatal. For this reason, the essential oil is generally avoided for internal use, although some elderly people consider it to be a very effective abortifacient. In addition, there are known cases of inhalation use, the Spanish in Mexico smoked *R. graveolens* mixed with tobacco in small quantities, probably to relieve headaches, nervous tension, or as a ritual practice (Conway and Slocumb 1979).

### 5.11 Morocco

In traditional Moroccan medicine, *R. graveolens* was widely used in various medicinal forms—infusions, decoctions, juice, ointments, powders, and vaginal enemas. Internally, the plant was used to treat digestive disorders, liver disease, migraines, nosebleeds, respiratory disorders, and more. Children were prescribed powder inhalations for fever and boiled herbs in milk to calm the nervous system. The juice of the fresh plant was used as eye drops for conjunctivitis, and decoctions from the flowers and roots were used in the form of vaginal enemas for abortion. Externally, *R. graveolens* was used in the form of ointments for rheumatism and vitiligo, as well as ear drops for tinnitus and infections. In addition, a mixture of plants, including *R. graveolens* , was fumigated for the treatment of epilepsy and liver diseases. Infusions were prescribed for colic, worms, and insect bites (Bellakhdar 1997).

### 5.12 Poland

In Polish local medicine, *R. graveolens* was traditionally used as a diuretic for edema (Olas et al. 2024). Poles believed that *R. graveolens* helped fight epilepsy, colds, fever, and cholera. It was also used for digestive problems and respiratory diseases, as well as externally for insect bites (Paluch 2002).

### 5.13 Republic of South Africa

In traditional South African medicine, especially in the Western Cape Province, *R. graveolens* is mainly used in the form of water infusions, decoctions, fresh juice, leaf teas, essential oils, as well as in the form of compresses and repellents. The infusion and decoction of the leaves were taken for urinary tract infections, bladder infections, and kidney problems (Chakale et al. 2024). The infusion was also taken for colds, to prevent fever, to treat headaches, and for stomach problems (De Beer and Van Wyk 2011). Juice squeezed from the leaves was used for infant convulsions, and the herb was used for nervous disorders (Masondo et al. 2019; Stafford et al. 2008). Tea made from the leaves was drunk for stomach pain, and bags of raw material were tied to the waist for disinfection and as a natural repellent (Van Wyk et al. 2008). Oil from the leaves of the plant was used to treat hysteria (Stafford et al. 2007). Tea made from the leaves of the plant was also used for various diseases of the genitourinary system, nervous disorders, various types of pain, and other conditions (Thring and Weitz 2006).

### 5.14 Romania

In traditional Romanian medicine, the plant was mainly used in the form of fresh juice, for example, three drops internally. It was used for fever, respiratory diseases to eliminate phlegm in the throat, and gastrointestinal disorders such as dysentery or aphthous stomatitis (Petran et al. 2020).

### 5.15 South America (Brazil)

In traditional South American medicine, particularly in Brazil, *R. graveolens* was widely used both as a monotherapy and as part of multi‐component herbal remedies. It was valued for its medicinal properties, including antispasmodic action, and was used for convulsions, digestive, renal, and nervous disorders, infectious diseases, as an anthelmintic, anti‐inflammatory, antipyretic, and antiseptic agent (Albuquerque et al. 2018; Cartaxo et al. 2010; Medeiros et al. 2013). In Brazilian cultural practice, *R. graveolens* has become particularly widespread in women's health, both for regulating the menstrual cycle and reducing menstrual pain, as well as for abortive purposes (De Freitas et al. 2005; Yazbek et al. 2016).

### 5.16 Taiwan

In traditional Taiwanese medicine, *R. graveolens* is commonly used to protect the heart and normalize heartbeat (Seak and Lin 2007).

### 5.17 Ukraine

*R. graveolens* is well known in traditional Ukrainian medicine and has a fairly wide range of indications (Colucci‐D'Amato and Cimaglia 2020). There are many recipes and methods of application. For example, for stomach pain, 10 drops of an alcohol tincture made from the leaves were taken three times a day. An aqueous extract mixed with other plants was used to improve appetite, three glasses per day. For nervous system disorders, combined teas with an aqueous extract of *R. graveolens* were used, 1 glass per day. The powder of the plant's herb with wine was taken for diseases of the urinary system and gallstone disease. A decoction of *R. graveolens* leaves with parsley was taken for complaints of painful and light menstruation. In addition, the raw material was widely used externally—in children's baths for scrofula and rickets, eyes were washed with an aqueous infusion, applied to wounds together with honey or in a crushed state on bruises and contusions, injected into the ear canal for hearing loss (Hammerman et al. 1990; Nosal and Nosal 2013).

### 5.18 Uruguay

In Uruguayan local medicine, the raw material was commonly used to treat inflammation, nervous disorders, arthritis, convulsions, skin diseases, fibromyalgia, as an antidote, and as a remedy for insects and other things (Ghosh et al. 2013; Koblovská et al. 2008). It was the most famous plant used for abortions in Uruguay (Ciganda and Laborde 2003).

## 6 Study of Chemical Composition

*R. graveolens* is distinguished by its particularly rich biochemical composition: a wide range of phenolic acids, flavonoids, coumarins, terpenes, and alkaloids have been identified in various plant organs and in vitro cultures. Thanks to the use of modern chromatographic and spectroscopic methods, it was possible not only to confirm the presence of known substances, but also to identify a number of new compounds that had not been described before. It should be noted that the numbering of all chemical compounds is unified and remains consistent throughout the entire manuscript, including the text, figures, and tables, to facilitate seamless cross‐referencing and ensure clarity.

### 6.1 Phenolic Compounds

In methanol and ethanol extracts of the aerial parts of *R. graveolens* , its roots, and in vitro cultures harvested in Portugal, Poland, the Czech Republic, Saudi Arabia, Egypt, Algeria, Mexico, and Central European countries, more than 25 phenolcarboxylic acid derivatives were identified, as shown in Figure 4 and in Table 2. The compounds were identified using chromatographic methods such as HPLC‐DAD, UPLC‐ESI‐.

**FIGURE 4:**
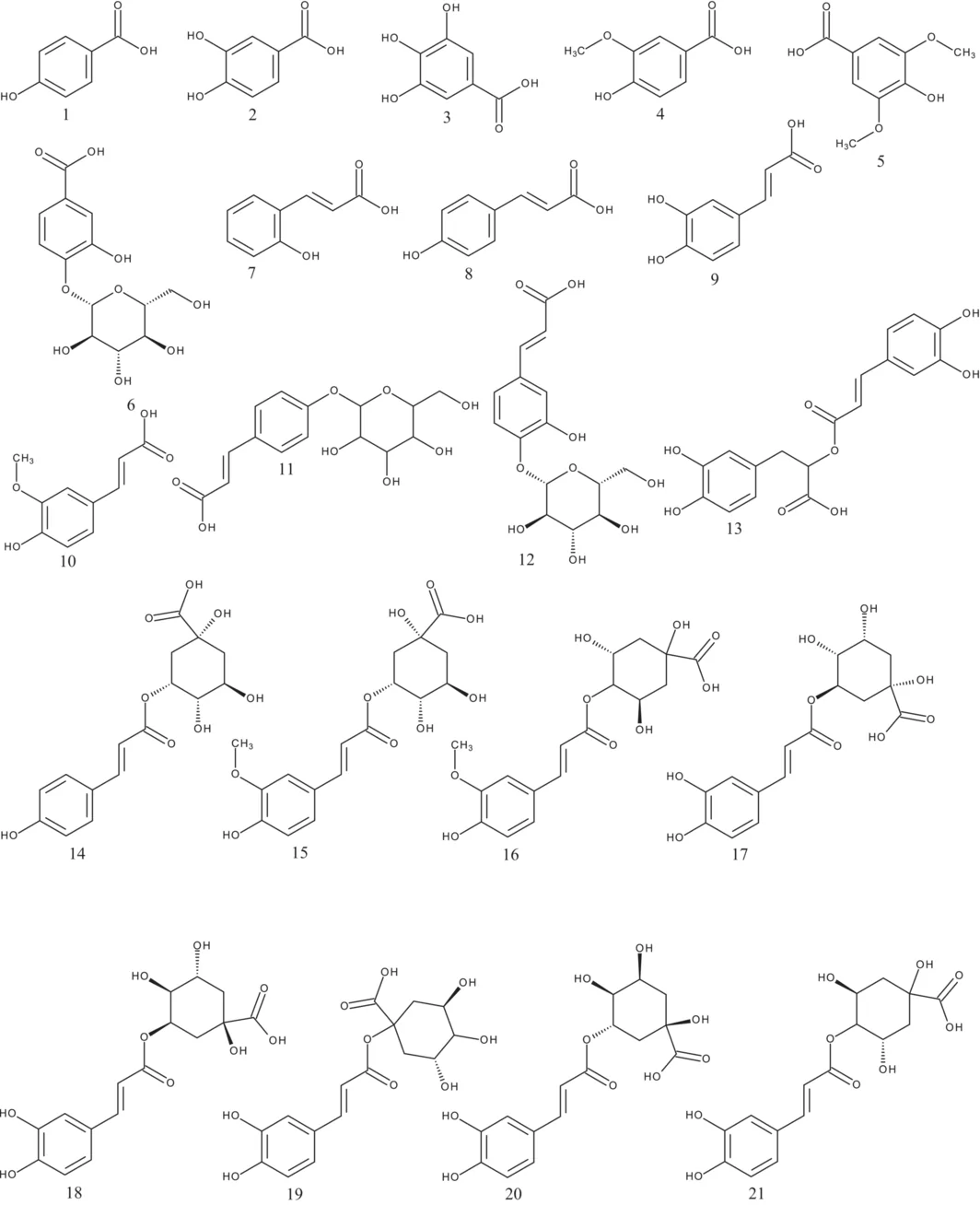
Phenolic compounds of *R. graveolens* L. This figure illustrates the structural diversity of phenolic acids and related derivatives established for the species, demonstrating the diversity of chemical skeletons.

**TABLE 2:** Compounds identified in *R. graveolens* raw material.

*N*	Compound	Plant part	Type of analysis	Plant origin	References
Phenolcarboxylic acids					
Hydroxybenzoic acids					
1	*p*‐Hydroxybenzoic acid	In vitro cultures	HPLC	Poland, Czech Republic	Szewczyk et al. 2023
2	Protocatechuic acid	Aerial parts	HPLC‐DAD‐ESI‐MS	Algeria	Mokhtar et al. 2022
		In vitro cultures	HPLC	Poland, Czech Republic	Szewczyk et al. 2023
3	Gallic acid	In vitro cultures	HPLC	Poland, Czech Republic	Szewczyk et al. 2023
4	Vanillic acid	Leaf and stem	HPLC–DAD	Portugal	Yu et al. 2021
5	Syringic acid	Aerial parts	UPLC‐ESI‐MS/MS	Kingdom of Saudi Arabia	Alyami et al. 2024
		Aerial parts	HPLC‐DAD‐ESI‐MS	Algeria	Mokhtar et al. 2022
		In vitro cultures	HPLC	Poland, Czech Republic	Szewczyk et al. 2023
6	Protocatechuic acid 4‐O‐glucoside	Leaves and shoots	HPLC–MS	Mexico	Tucuch‐Pérez et al. 2023
Hydroxycinnamic acids and their derivatives					
7	*o*‐Coumaric acid	Leaf and stem	HPLC–DAD	Portugal	Yu et al. 2021
8	*р*‐Coumaric acid	Herb	HPLC	Not indicated	Melnyk et al. 2018
		In vitro cultures	HPLC	Poland, Czech Republic	Szewczyk et al. 2023
9	Caffeic acid	Leaves	СС, ^1^H‐, ^13^CNMR	Egypt	Salib et al. 2014
		Herb	HPLC	Not indicated	Melnyk et al. 2018
10	Ferulic acid	Leaves	СС, ^1^H‐, ^13^C NMR	Egypt	Salib et al. 2014
		In vitro cultures	HPLC	Poland, Czech Republic	Szewczyk et al. 2023
11	*p*‐Coumaric acid 4‐O‐glucoside	Leaves and shoots	HPLC–MS	Mexico	Tucuch‐Pérez et al. 2023
12	Caffeic acid 4‐O‐glucoside	Leaves and shoots	HPLC–MS	Mexico	Tucuch‐Pérez et al. 2023
13	Rosmarinic acid	Aerial parts	UPLC‐ESI‐MS/MS	Kingdom of Saudi Arabia	Alyamiet al., 2024
		Herb	HPLC	Not indicated	Melnyk et al. 2018
14	3‐p‐Coumaroylquinic acid	Leaves and shoots	HPLC–MS	Mexico	Tucuch‐Pérez et al. 2023
15	3‐Feruloylquinic acid	Leaves and shoots	HPLC–MS	Mexico	Tucuch‐Pérez et al. 2023
16	4‐Feruloylquinic acid	Leaves and shoots	HPLC‐MS	Mexico	Tucuch‐Pérez et al. 2023
17	Chlorogenic acid	Leaf and stem	HPLC–DAD	Portugal	Yu et al. 2021
		Herb	HPLC	Not indicated	Melnyk et al. 2018
18	Neochlorogenic Acid	Leaf and stem	HPLC–DAD	Portugal	Yu et al. 2021
		Aerial parts	UPLC‐ESI‐MS/MS	Kingdom of Saudi Arabia	Alyami et al. 2024
19	1‐Caffeoylquinic acid	Leaves and shoots	HPLC‐MS	Mexico	Tucuch‐Pérez et al. 2023
20	3‐Caffeoylquinic acid	Aerial parts	UPLC‐ESI‐MS/MS	Kingdom of Saudi Arabia	Alyami et al. 2024
21	4‐Caffeoylquinic acid	Aerial parts	UPLC‐ESI‐MS/MS	Kingdom of Saudi Arabia	Alyami et al. 2024
Flavonoids					
Flavan‐3‐ols					
22	Epicatechin	Aerial parts	UPLC‐ESI‐MS/MS	Kingdom of Saudi Arabia	Alyami et al. 2024
23	Gallocatechin	Leaf and stem	HPLC–DAD	Portugal	Yu et al. 2021
24	Epicatechin gallate	Leaf and stem	HPLC–DAD	Portugal	Yu et al. 2021
Flavanones					
25	Naringenin	Aerial parts	HPLC‐DAD‐ESI‐MS	Algeria	Mokhtar et al. 2022
Flavones and their glycosides					
26	Chrysin	Aerial parts	HPLC‐DAD‐ESI‐MS	Algeria	Mokhtar et al. 2022
27	Apigenin	Leaf and stem	HPLC–DAD	Portugal	Yu et al. 2021
		Aerial parts	HPLC	Iran	Asgharian et al. 2020
		Aerial parts	HPLC‐DAD‐ESI‐MS	Algeria	Mokhtar et al. 2022
		Herb	HPLC	Not indicated	Melnyk et al. 2018
28	Luteolin	Herb	HPLC	Not indicated	Melnyk et al. 2018
29	Quercetin	Leaves	HPLC	Brazil	Cefali et al. 2019
		Leaves and aerial parts	LC‐MS MS	Temuco in Southern Chile	Águila et al. 2015
		Leaves	СС, ^1^H NMR, ^13^C NMR	Egypt	Salib et al. 2014
		Aerial parts	UPLC‐ESI‐MS/MS	Kingdom of Saudi Arabia	Alyami et al. 2024
		Herb	HPLC	Not indicated	Melnyk et al. 2018
30	Isorhamnetin	Leaves	СС, ^1^H NMR, ^13^C NMR	Egypt	Salib et al. 2014
31	Kaempferol	Leaves	СС, ^1^H NMR, ^13^C NMR	Egypt	Salib et al. 2014
32	Isoquercitrin (Isoquercetin, Quercetin‐3‐O‐β‐D‐glucopyranoside)	Aerial parts	UPLC‐ESI‐MS/MS	Kingdom of Saudi Arabia	Alyami et al. 2024
		Leaves	СС, ^1^H NMR, ^13^C NMR	Egypt	Salib et al. 2014
		Aerial parts	HPLC‐DAD‐ESI‐MS	Algeria	Mokhtar et al. 2022
		Herb	HPLC	Not indicated	Melnyk et al. 2018
33	Astragalin	Leaves	СС, ^1^H NMR, ^13^C NMR	Egypt	Salib et al. 2014
34	Hyperoside	Aerial parts	HPLC‐DAD‐ESI‐MS	Algeria	Mokhtar et al. 2022
35	Quercetin‐3‐O‐*α*‐L‐rhamnopyranoside	Leaves	СС, ^1^H NMR, ^13^C NMR	Egypt	Salib et al. 2014
36	Rutin	Leaf and stem	HPLC–DAD	Portugal	Yu et al. 2021
		Aerial parts	HPLC	Iran	Asgharian et al. 2020
		Leaves	HPLC	Brazil	Cefali et al. 2019
		Leaves and aerial parts	LC‐MS MS	Temuco in Southern Chile	Águila et al. 2015
		Aerial parts	UPLC‐ESI‐MS/MS	Kingdom of Saudi Arabia	Alyami et al. 2024
		Aerial parts	HPLC‐DAD‐ESI‐MS	Algeria	Mokhtar et al. 2022
		Herb	HPLC	Not indicated	Melnyk et al. 2018
		Leaves	СС, ^1^H NMR, ^13^C NMR	Egypt	Salib et al. 2014
37	Quercetin 3‐O‐rhamnosyl‐galactoside	Leaves and shoots	HPLC‐MS	Mexico	Tucuch‐Pérez et al. 2023
38	Narcissin	Aerial parts	UPLC‐ESI‐MS/MS	Kingdom of Saudi Arabia	Alyami et al. 2024
39	Quercetin 3‐O‐xylosyl‐glucuronide	Leaves and shoots	HPLC‐MS	Mexico	Tucuch‐Pérez et al. 2023
40	Vicenin‐2	Aerial parts	UPLC‐ESI‐MS/MS	Kingdom of Saudi Arabia	Alyami et al. 2024
Coumarins					
Simple coumarins					
41	Umbelliferone	Aerial parts	UPLC‐ESI‐MS/MS	Kingdom of Saudi Arabia	Alyami et al. 2024
42	7‐Methoxycoumarin	Aerial parts	GC–q‐TOF‐MS	Mexico	Reyes‐Vaquero et al. 2024
43	8‐Methoxy‐chromen‐2‐one	Whole plant	HPLC	India	Sahu et al. 2015
44	5,7‐Dimethoxycoumarin	Aerial parts	GC–q‐TOF‐MS	Mexico	Reyes‐Vaquero et al. 2024
45	6,7,8‐Trimethoxycoumarin	Roots	GC–q‐TOF‐MS	Mexico	Reyes‐Vaquero et al. 2024
46	Scopoletin	Aerial parts	UPLC‐ESI‐MS/MS	Kingdom of Saudi Arabia	Alyami et al. 2024
		Aerial parts and shoots	GC–MS	Not indicated	Naghibi Harat et al. 2015
47	Gravelliferone*	Aerial parts	UPLC‐ESI‐MS/MS	Kingdom of Saudi Arabia	Alyami et al. 2024
48	3‐(1,1‐Dimethylallyl)‐7,8‐dimethoxycoumarin	Leaves, roots	CC, HPLC	Japan	Nakano et al. 2016
49	3‐(1,1‐Dimethylallyl) herniarin	Aerial parts	UPLC‐ESI‐MS/MS	Kingdom of Saudi Arabia	Alyami et al. 2024
50	Ostol	Aerial parts, roots	GC–q‐TOF‐MS	Mexico	Reyes‐Vaquero et al. 2024
51	3‐(1,1‐DimethylaUyl)‐6‐(2‐hydroxy‐3‐methyl‐3‐buten‐l‐yl)‐7‐methoxycoumarin	Leaves, roots	CC, HPLC	Japan	Nakano et al. 2016
52	3‐(14‐DimethylaUyl)‐6‐(2‐hydroxy‐3‐methyl‐3‐buten‐l‐yl)‐7‐hydroxycoumarin	Leaves, roots	CC, HPLC	Japan	Nakano et al. 2016
53	3‐(1,1‐Dimethylallyl) scopoletin	Roots	GC–q‐TOF‐MS	Mexico	Reyes‐Vaquero et al. 2024
54	Murralongin*	Leaves, roots	CC, HPLC	Japan	Nakano et al. 2016
55	Rutacycoumarin A*	Aerial parts	NMR, HPLC	China	Feng et al. 2026
56	Rutacycoumarin B*	Aerial parts	NMR, HPLC	China	Feng et al. 2026
57	Graveoumarin A*	Aerial parts	NMR, HPLC	China	Wu et al. 2026
58	Graveoumarin B*	Aerial parts	NMR, HPLC	China	Wu et al. 2026
59	Graveoumarin C*	Aerial parts	NMR, HPLC	China	Wu et al. 2026
Counarin dimers and their ethers					
60	Daphnoretin	Aerial parts	UPLC‐ESI‐MS/MS	Kingdom of Saudi Arabia	Alyami et al. 2024
61	Daphnoretin methyl ether	Aerial parts	UPLC‐ESI‐MS/MS	Kingdom of Saudi Arabia	Alyami et al. 2024
Furocoumarins					
62	Psoralen	Leaves, roots	CC, HPLC	Japan	Nakano et al. 2016
		Aerial parts	UPLC‐ESI‐MS/MS	Kingdom of Saudi Arabia	Alyami et al. 2024
		Vitro cultures	HPLC	Poland, Czech Republic	Szewczyk et al. 2023
		In vitro shoot culture	HPLC	Poland, Czech Republic	Szewczyk et al. 2022
		Aerial parts and shoots	GC–MS	Not indicated	Naghibi Harat et al. 2015
		Aerial parts, roots	GC–q‐TOF‐MS	Mexico	Reyes‐Vaquero et al. 2024
		Aerial parts	GC–MS	Mexico	Reyes‐Vaquero et al. 2021
63	Bergapten	Leaves, roots	CC, HPLC	Japan	Nakano et al. 2016
		In vitro cultures	HPLC	Poland, Czech Republic	Szewczyk et al. 2023
		In vitro shoot culture	HPLC	Poland, Czech Republic	Szewczyk et al. 2022
		Aerial parts, roots	GC–q‐TOF‐MS	Mexico	Reyes‐Vaquero et al. 2024
		Aerial parts and shoots	GC–MS	Not indicated	Naghibi Harat et al. 2015
64	Xanthotoxin	Leaves, roots	CC, HPLC	Japan	Nakano et al. 2016
		Aerial parts	UPLC‐ESI‐MS/MS	Kingdom of Saudi Arabia	Alyami et al. 2024
		In vitro cultures	HPLC	Poland, Czech Republic	Szewczyk et al. 2023
		In vitro shoot culture	HPLC	Poland, Czech Republic	Szewczyk et al. 2022
		Roots	GC–q‐TOF‐MS	Mexico	Reyes‐Vaquero et al. 2024
		Aerial parts and shoots	GC–MS	Not indicated	Naghibi Harat et al. 2015
		Roots	GC–MS	Mexico	Reyes‐Vaquero et al. 2021
65	Isopimpinelin*	Leaves, roots	CC, HPLC	Japan	Nakano et al. 2016
		In vitro cultures	HPLC	Poland, Czech Republic	Szewczyk et al. 2023
		In vitro shoot culture	HPLC	Poland, Czech Republic	Szewczyk et al. 2022
		Aerial parts, roots	GC–q‐TOF‐MS	Mexico	Reyes‐Vaquero et al. 2024
		Roots	GC–MS	Mexico	Reyes‐Vaquero et al. 2021
66	Isoimperatorin*	In vitro cultures	HPLC	Poland, Czech Republic	Szewczyk et al. 2023
		In vitro shoot culture	HPLC	Poland, Czech Republic	Szewczyk et al. 2022
67	Benahorin*	Roots	GC–q‐TOF‐MS	Mexico	Reyes‐Vaquero et al. 2024
68	Chalepensin*	Aerial parts	UPLC‐ESI‐MS/MS	Kingdom of Saudi Arabia	Alyami et al. 2024
		Leaves, roots	CC, HPLC	Japan	Nakano et al. 2016
		Aerial parts, roots	GC–q‐TOF‐MS	Mexico	Reyes‐Vaquero et al. 2024
Dihydrofurocoumarins and their derivatives					
69	Chalepin*	Aerial parts	UPLC‐ESI‐MS/MS	Kingdom of Saudi Arabia	Alyamiet al. 2024
		Aerial parts, roots	GC–q‐TOF‐MS	Mexico	Reyes‐Vaquero et al. 2024
		Aerial parts	CC, ^1^H NMR, ^13^C NMR, EIMS	China	Lin et al. 2017
70	Rutamarin*	Leaves, roots	CC, HPLC	Japan	Nakano et al. 2016
		Aerial parts, roots	GC–q‐TOF‐MS	Mexico	Reyes‐Vaquero et al. 2024
		Aerial parts	UPLC‐ESI‐MS/MS	Kingdom of Saudi Arabia	Alyami et al. 2024
		Aerial parts	HPLC	Poland	Kozioł et al. 2020
71	Rutaretin*	Leaves, roots	CC, HPLC	Japan	Nakano et al. 2016
		Aerial parts	UPLC‐ESI‐MS/MS	Kingdom of Saudi Arabia	Alyami et al. 2024
72	Heraclenol	Leaves, roots	CC, HPLC	Japan	Nakano et al. 2016
73	Rutarin*	Leaves, roots	CC, HPLC	Japan	Nakano et al. 2016
Pyranocoumarins					
74	Seselin	Roots	GC–q‐TOF‐MS	Mexico	Reyes‐Vaquero et al. 2024
75	Xanthyletin	Leaves, roots	CC, HPLC	Japan	Nakano et al. 2016
Other					
76	Rutabenzofuran A*	Aerial parts	^1^H NMR, ^13^C NMR, HR‐ESI‐MS, HPLC	China	Liu et al. 2023
77	Rutabenzofuran B*	Aerial parts	^1^H NMR, ^13^C NMR, HR‐ESI‐MS, HPLC	China	Liu et al. 2023
Terpenes					
Monoterpenoids					
78	Citronellal	Roots	GC–q‐TOF‐MS	Mexico	Reyes‐Vaquero et al. 2024
79	α‐Limonene	Aerial parts (essential oil)	GC–MS	Algeria (Anaba) ‐ a‐Limonene, Tunisia (Bizerta) – Limonene	Nahar et al. 2021
80	p‐Mentha‐1,5,8‐triene	Roots	GC–q‐TOF‐MS	Mexico	Reyes‐Vaquero et al. 2024
81	para‐Cymene	Aerial parts (essential oil)	GC–MS	Romania	Jianu et al. 2021
		Aerial parts (essential oil)	GC–MS	Tunisia	Chaftar et al. 2015
82	m‐Cymenene	Aerial parts and roots	GC–q‐TOF‐MS	Mexico	Reyes‐Vaquero et al. 2024
83	Carvacrol	Essential oil	GC–MS	Not indicated	D'Addabbo and Avato 2021
84	Pulegone	Aerial parts	GC–q‐TOF‐MS	Mexico	Reyes‐Vaquero et al. 2024
85	Carvenone	Aerial parts	GC–q‐TOF‐MS	Mexico	Reyes‐Vaquero et al. 2024
86	2‐Bornene	Aerial parts (essential oil)	GC–MS	Romania	Jianu et al. 2021
87	Borneol	Aerial parts (essential oil)	GC–MS	Tunisia	Chaftar et al. 2015
88	Camphor	Aerial parts (essential oil)	GC–MS	Tunisia	Chaftar et al. 2015
89	4‐Carene	Aerial parts (essential oil)	GC–MS	Romania	Jianu et al. 2021
90	trans‐3‐Caren‐2‐ol	Aerial parts	GC–q‐TOF‐MS	Mexico	Reyes‐Vaquero et al. 2024
91	trans‐3(10)‐Caren‐2‐ol	Aerial parts	GC–q‐TOF‐MS	Mexico	Reyes‐Vaquero et al. 2024
92	trans‐Verbenol	Aerial parts	GC–q‐TOF‐MS	Mexico	Reyes‐Vaquero et al. 2024
93	Sabinene	Roots	GC–q‐TOF‐MS	Mexico	Reyes‐Vaquero et al. 2024
94	β‐Thujene	Aerial parts (essential oil)	GC–MS	Romania	Jianu et al. 2021
		Aerial parts (essential oil)	GC–MS	Tunisia	Chaftar et al. 2015
95	α‐Thujon	Aerial parts (essential oil)	GC–MS	Tunisia	Chaftar et al. 2015
96	Chrysanthenone	Aerial parts (essential oil)	GC–MS	Tunisia	Chaftar et al. 2015
97	β‐Terpinyl acetate	Aerial parts (essential oil)	GC–MS	Romania	Jianu et al. 2021
98	Eucalyptol	Aerial parts (essential oil)	GC–MS	Romania	Jianu et al. 2021
		Aerial parts (essential oil)	GC–MS	Tunisia	Chaftar et al. 2015
99	Isoascaridol	Aerial parts and roots	GC–q‐TOF‐MS	Mexico	Reyes‐Vaquero et al. 2024
100	p‐Mentha‐1(7),8‐dien‐2‐ol	Roots	GC–q‐TOF‐MS	Mexico	Reyes‐Vaquero et al. 2024
101	p‐Mentha‐2,8‐dien‐1‐ol	Aerial parts and roots	GC–q‐TOF‐MS	Mexico	Reyes‐Vaquero et al. 2024
102	trans‐Carveol	Aerial parts and roots	GC–q‐TOF‐MS	Mexico	Reyes‐Vaquero et al. 2024
103	(S)‐(+)‐Carvone	Aerial parts (essential oil)	GC–MS	Romania	Jianu et al. 2021
104	trans‐Ascaridolglycol	Aerial parts and roots	GC–q‐TOF‐MS	Mexico	Reyes‐Vaquero et al. 2024
105	Limonen‐6‐ol, pivalate	Aerial parts	GC–q‐TOF‐MS	Mexico	Reyes‐Vaquero et al. 2024
Sesquiterpenes					
106	α‐Farnesene	Aerial parts (essential oil)	prep‐GC, GC‐QTOF‐MS, NMR	China	Li et al. 2020
107	(Z,E)‐α‐Farnesene	Aerial parts (essential oil)	prep‐GC, GC‐QTOF‐MS, NMR	China	Li et al. 2020
108	Hexa‐hydro‐farnesol	Aerial parts (essential oil)	GC–MS	Romania	Jianu et al. 2021
109	Calarene	Aerial parts	GC–q‐TOF‐MS	Mexico	Reyes‐Vaquero et al. 2024
110	β‐caryophyllene	Aerial parts (essential oil)	GC–MS	Peru	Nahar et al. 2021
		Aerial parts (essential oil)	GC–MS	Romania	Jianu et al. 2021
111	Geijerene*	Aerial parts (essential oil)	prep‐GC, GC‐QTOF‐MS, NMR	China	Li et al. 2020
112	Elemol	Aerial parts (essential oil)	GC–MS	Romania	Jianu et al. 2021
113	Germacrene‐D	Aerial parts (essential oil)	GC–MS	Romania	Jianu et al. 2021
114	α‐Eudesmol	Aerial parts (essential oil)	GC–MS	Romania	Jianu et al. 2021
115	Cedrol	Aerial parts (essential oil)	GC–MS	Tunisia	Chaftar et al. 2015
116	Corymbolone	Aerial parts (essential oil)	GC–MS	Romania	Jianu et al. 2021
Other					
117	Eugenol	Essential oil	GC–MS	Not indicated	D'Addabbo and Avato 2021
118	α‐Ionone	Roots	GC–q‐TOF‐MS	Mexico	Reyes‐Vaquero et al. 2024
119	trans‐β‐Ionone	Roots	GC–q‐TOF‐MS	Mexico	Reyes‐Vaquero et al. 2024
Alkaloids					
Quinoline alkaloids					
120	N‐methyl‐4‐methoxy‐2‐quinolone*	Leaves	CC	Not indicated	Sampaio et al. 2018
121	3‐Methoxy‐l‐methyl‐2‐qunolinone	Roots	CC, HPLC	Japan	Nakano et al. 2016
122	2‐heptyl‐4(1H)quinolone	Aerial parts	CC	China	Ainiwaer et al. 2023
123	2‐Nonyl‐4(lH)‐quinolone	Leaves	CC, HPLC	Japan	Nakano et al. 2016
124	1‐methyl‐2‐nonyl‐4(1H)‐ quinolone	Aerial parts	UPLC‐ESI‐QTOF‐MS	Brazil	Carvalho et al. 2019
		Aerial parts	UPLC‐ESI‐MS/MS	Kingdom of Saudi Arabia	Alyami et al. 2024
		Leaves	CC, HPLC	Japan	Nakano et al. 2016
125	1‐methyl‐2‐decyl‐4(1H)‐ quinolone	Aerial parts	UPLC‐ESI‐QTOF‐MS	Brazil	Carvalho et al. 2019
		Aerial parts	UPLC‐ESI‐MS/MS	Kingdom of Saudi Arabia	Alyami et al. 2024
126	1‐methyl‐2‐undecyl‐4(1H)‐ quinolone	Aerial parts	UPLC‐ESI‐QTOF‐MS	Brazil	Carvalho et al. 2019
		Aerial parts	UPLC‐ESI‐MS/MS	Kingdom of Saudi Arabia	Alyami et al. 2024
127	2‐Methyl‐3‐undecyl‐1H‐quinolin‐4‐one	Roots	GC–q‐TOF‐MS	Mexico	Reyes‐Vaquero et al. 2024
128	1‐methyl‐2‐dodecyl‐4(1H)‐ quinolone	Aerial parts	UPLC‐ESI‐QTOF‐MS	Brazil	Carvalho et al. 2019
		Aerial parts	UPLC‐ESI‐MS/MS	Kingdom of Saudi Arabia	Alyami et al. 2024
129	Dihydroevocarpine	Aerial parts	UPLC‐ESI‐QTOF‐MS	Brazil	Carvalho et al. 2019
130	3‐Methyl‐2‐nonyl‐1H‐quinolin‐4‐one	Roots	GC–q‐TOF‐MS	Mexico	Reyes‐Vaquero et al. 2024
131	3‐Methyl‐2‐pentyl‐1H‐quinolin‐4‐one	Roots	GC–q‐TOF‐MS	Mexico	Reyes‐Vaquero et al. 2024
132	3‐Methyl‐2‐undecyl‐1H‐quinolin‐4‐one	Roots	GC–q‐TOF‐MS	Mexico	Reyes‐Vaquero et al. 2024
133	2‐n‐heptyl‐4‐hydroxyquinoline	Aerial parts	CC	China	Ainiwaer et al. 2023
134	4‐hydroxy‐2‐decylquinoline	Aerial parts	UPLC‐ESI‐QTOF‐MS	Brazil	Carvalho et al. 2019
		Aerial parts	UPLC‐ESI‐MS/MS	Kingdom of Saudi Arabia	Alyami et al. 2024
135	4‐hydroxy‐2‐ undecylquinoline	Aerial parts	UPLC‐ESI‐QTOF‐MS	Brazil	Carvalho et al. 2019
136	Graveoline (rutamine)*	Leaves	CC, HPLC	Japan	Nakano et al. 2016
		Leaves	CC	Not indicated	Sampaio et al. 2018
		Aerial parts	CC	China	Ainiwaer et al. 2023
		Aerial parts	UPLC‐ESI‐QTOF‐MS	Brazil	Carvalho et al. 2019
		Aerial parts	UPLC‐ESI‐MS/MS	Kingdom of Saudi Arabia	Alyami et al. 2024
		Roots	GC–q‐TOF‐MS, GC–q‐TOF‐MS	Mexico	Reyes‐Vaquero et al. 2024
137	2‐(1,3‐Benzodioxol‐5‐yl)butyl‐4(1H)‐quinolinone	Leaves	CC, HPLC	Japan	Nakano et al. 2016
138	2‐(1,3‐Benzodioxol‐5‐yl)butyl‐l‐methyl‐4(1H)‐quinolinone	Leaves	CC, HPLC	Japan	Nakano et al. 2016
139	2‐(1,3‐Benzodioxol‐5‐yl)hexyl‐l‐methyl −4(1H)‐quinolinone	Leaves	CC, HPLC	Japan	Nakano et al. 2016
140	2‐[4′‐(3′,4′‐methylenedioxyphenyl)butyl]‐4‐quinolone	Aerial parts	CC	China	Ainiwaer et al. 2023
141	Graveolinine*	Aerial parts	UPLC‐ESI‐QTOF‐MS	Brazil	Carvalho et al. 2019
		Aerial parts	UPLC‐ESI‐MS/MS	Kingdom of Saudi Arabia	Alyami et al. 2024
Furoquinoline alkaloids					
142	Dictamnine*	Roots	CC, HPLC	Japan	Nakano et al. 2016
		Aerial parts	UPLC‐ESI‐MS/MS	Kingdom of Saudi Arabia	Alyami et al. 2024
143	Pteleine*	Aerial parts	UPLC‐ESI‐MS/MS	Kingdom of Saudi Arabia	Alyami et al. 2024
		Aerial parts, roots	GC–q‐TOF‐MS	Mexico	Reyes‐Vaquero et al. 2024
144	γ –fagarine (8‐Methoxydictamnine)*	In vitro shoot culture	HPLC	Poland, Czech Republic	Szewczyk et al. 2022
		Leaves	CC, HPLC	Japan	Nakano et al. 2016
		In vitro cultures	HPLC	Poland, Czech Republic	Szewczyk et al. 2023
		Aerial parts	UPLC‐ESI‐MS/MS	Kingdom of Saudi Arabia	Alyami et al. 2024
145	Kokusaginine*	Aerial parts	CC	China	Ainiwaer et al. 2023
		Aerial parts	HPLC	Italy	Mancuso et al. 2015
		Aerial parts	UPLC‐ESI‐MS/MS	Kingdom of Saudi Arabia	Alyami et al. 2024
		Aerial parts, roots	GC–q‐TOF‐MS	Mexico	Reyes‐Vaquero et al. 2024
146	Skimmianine (β‐fagarine)*	Aerial parts	CC	China	Ainiwaer et al. 2023
		In vitro shoot culture	HPLC	Poland, Czech Republic	Szewczyk et al. 2022
		Leaves	CC, HPLC	Japan	Nakano et al. 2016
		Aerial parts	HPLC	Brazil	Mancuso et al. 2015
		Vitro cultures	HPLC	Poland, Czech Republic	Szewczyk et al. 2023
		Aerial parts	UPLC‐ESI‐QTOF‐MS	Brazil	Carvalho et al. 2019
		Aerial parts	UPLC‐ESI‐MS/MS	Kingdom of Saudi Arabia	Alyamiet al., 2024
		Aerial parts, roots	GC–q‐TOF‐MS	Mexico	Reyes‐Vaquero et al. 2024
		Aerial parts	GC–MS	Mexico	Reyes‐Vaquero et al. 2021
147	Isopentenyloxy‐γ‐fagarine*	In vitro shoot culture	HPLC	Poland, Czech Republic	Szewczyk et al. 2022
		Vitro cultures	HPLC	Poland, Czech Republic	Szewczyk et al. 2023
148	(*4S*) 1,4‐dihydro‐4‐methoxy‐1,4‐dimethyl‐3‐(3‐methylbut‐2‐enyl)quinoline 2,7‐diol	Leaves	СС, ^1^H NMR, ^13^C NMR	Egypt	Salib et al. 2014
		Aerial parts	UPLC‐ESI‐MS/MS	Kingdom of Saudi Arabia	Alyami et al. 2024
149	(±)‐Ribaline*	Aerial parts	CC	China	Ainiwaer et al. 2023
150	Rutagrarin A*	Aerial parts	CC	China	Ainiwaer et al. 2023
Acridone alkaloids					
151	1‐Hydroxy‐10‐methylacridone	Roots	CC, HPLC	Japan	Nakano et al. 2016
		Roots	GC–q‐TOF‐MS	Mexico	Reyes‐Vaquero et al. 2024
		Roots	GC–MS	Mexico	Reyes‐Vaquero et al. 2021
152	1‐Hydroxy‐3‐methoxy‐10‐methyl‐9(10H)‐acridinone	Roots	GC–q‐TOF‐MS	Mexico	Reyes‐Vaquero et al. 2024
153	Arborinine*	Leaves	CC	Not indicated	Sampaio et al. 2018
		Aerial parts	CC	China	Ainiwaer et al. 2023
		Aerial parts	UPLC‐ESI‐QTOF‐MS	Brazil	Carvalho et al. 2019
		Aerial parts	UPLC‐ESI‐MS/MS	Kingdom of Saudi Arabia	Alyami et al. 2024
		Aerial parts, roots	GC–q‐TOF‐MS	Mexico	Reyes‐Vaquero et al. 2024
		Aerial parts	GC–MS	Mexico	Reyes‐Vaquero et al. 2021
154	1,3‐dihydroxy‐2‐methoxy‐10‐methyl‐9‐acridone	Aerial parts	CC	China	Ainiwaer et al. 2023
155	1‐hydroxy‐3‐methoxy‐N‐methylacridone	Leaves	CC	Not indicated	Sampaio et al. 2018
156	1,4‐dihydroxy‐2,3‐dimethoxy‐N‐methylacridone	Leaves	CC	Not indicated	Sampaio et al. 2018
157	Furofoline I*	Roots	GC–q‐TOF‐MS	Mexico	Reyes‐Vaquero et al. 2024
Furoacridone alkaloids					
158	Gravacridondiol*	Aerial parts	CC	China	Ainiwaer et al. 2023
		Aerial parts	UPLC‐ESI‐MS/MS	Kingdom of Saudi Arabia	Alyami et al. 2024
159	Gravacridonol*	Aerial parts	UPLC‐ESI‐MS/MS	Kingdom of Saudi Arabia	Alyami et al. 2024
160	Gravacridonetriol*	Aerial parts	UPLC‐ESI‐MS/MS	Kingdom of Saudi Arabia	Alyami et al. 2024
161	Rutacridone*	Roots	CC, HPLC	Japan	Nakano et al. 2016
		Aerial parts	UPLC‐ESI‐MS/MS	Kingdom of Saudi Arabia	Alyami et al. 2024
		Roots	GC–MS	Mexico	Reyes‐Vaquero et al. 2021
		Aerial parts, roots	GC–q‐TOF‐MS	Mexico	Reyes‐Vaquero et al. 2024
162	Rutacridone epoxide*	Aerial parts	UPLC‐ESI‐MS/MS	Kingdom of Saudi Arabia	Alyami et al. 2024
Pyrazine and other nitrogen‐containing heterocycles					
163	Xylogranatinin	Aerial parts	CC	China	Ainiwaer et al. 2023
164	Ribalinidine*	Leaves	CC, HPLC	Japan	Nakano et al. 2016
Glycosylated alkaloids and other					
165	Gravacridondiol‐18‐β‐D‐glucoside*	Aerial parts	CC	China	Ainiwaer et al. 2023
		Roots	CC, HPLC	Japan	Nakano et al. 2016

*Note:* Compounds marked with an asterisk (*) are characteristic metabolites of *R. graveolens* and related species within the Rutaceae family.

Abbreviations: CC, Column Chromatography; EIMS, Electron Ionization Mass Spectrometry; GC–MS, Gas Chromatography–Mass Spectrometry; GC–q‐TOF‐MS, Gas Chromatography–Quadrupole Time‐of‐Flight Mass Spectrometry; HPLC, High‐Performance Liquid Chromatography; HPLC–DAD, High‐Performance Liquid Chromatography with Diode Array Detection; HPLC‐DAD‐ESI‐MS, High‐Performance Liquid Chromatography with Diode Array Detection and Electrospray Ionization Mass Spectrometry; HPLC–MS, High‐Performance Liquid Chromatography–Mass Spectrometry; HR‐ESI‐MS, High‐Resolution Electrospray Ionization Mass Spectrometry; LC‐MS, Liquid Chromatography–Tandem Mass Spectrometry; NMR, Nuclear Magnetic Resonance (^1^H and ^13^C); prep‐GC, Preparative Gas Chromatography; UPLC‐ESI‐MS/MS, Ultra‐Performance Liquid Chromatography–Electrospray Ionization–Tandem Mass Spectrometry; UPLC‐ESI‐QTOF‐MS, Ultra‐Performance Liquid Chromatography–Electrospray Ionization–Quadrupole Time‐of‐Flight Mass Spectrometry.

Among hydroxybenzoic acids in *R. graveolens* raw materials, p‐hydroxybenzoic acid **1**, protocatechuic acid **2**, gallic acid **3**, vanillic acid **4**, syringic acid **5**, and protocatechuic acid 4‐O‐glucoside **6** were identified. Among hydroxycinnamic acids and their derivatives, numerous phenolic derivatives were found, including p‐coumaric acid **8**, caffeic acid **9**, ferulic acid **10**, rosmarinic acid **13**, chlorogenic acid **17**, and their glycosides. Similar compounds have been identified in raw material samples from different regions of the world using modern analytical methods.

A separate group consists of methoxycinnamic acids and their esters, such as 3‐feruloylquinic acid **15** and 4‐feruloylquinic acid **16**, which are found mainly in leaves and roots, using HPLC‐MS (Tucuch‐Pérez et al. 2023).

### 6.2 Flavonoids

Similar to hydroxycinnamic acids, flavonoids were also identified in *R. graveolens* raw materials of various geographical origins (Table 2). A total of about 15 flavonoid aglycones and their glycosides are described, which are presented in Figure 5. Among the main representatives of flavones, identified mainly in the aerial parts of *R. graveolens* , chrysin **26**, apigenin **27**, luteolin **28**, quercetin **29**, and others, as well as quercetin derivatives, in particular quercetin 3‐O‐rhamnosyl‐galactoside **37** and quercetin‐3‐O‐xylosyl‐glucuronide **39**. In addition, the rare C‐glycoside of apigenin vicenin‐2 **40** (Alyami et al. 2024), which has not been described for other species of *R. graveolens* , was identified in the aerial part of *R. graveolens* from Saudi Arabia.

**FIGURE 5:**
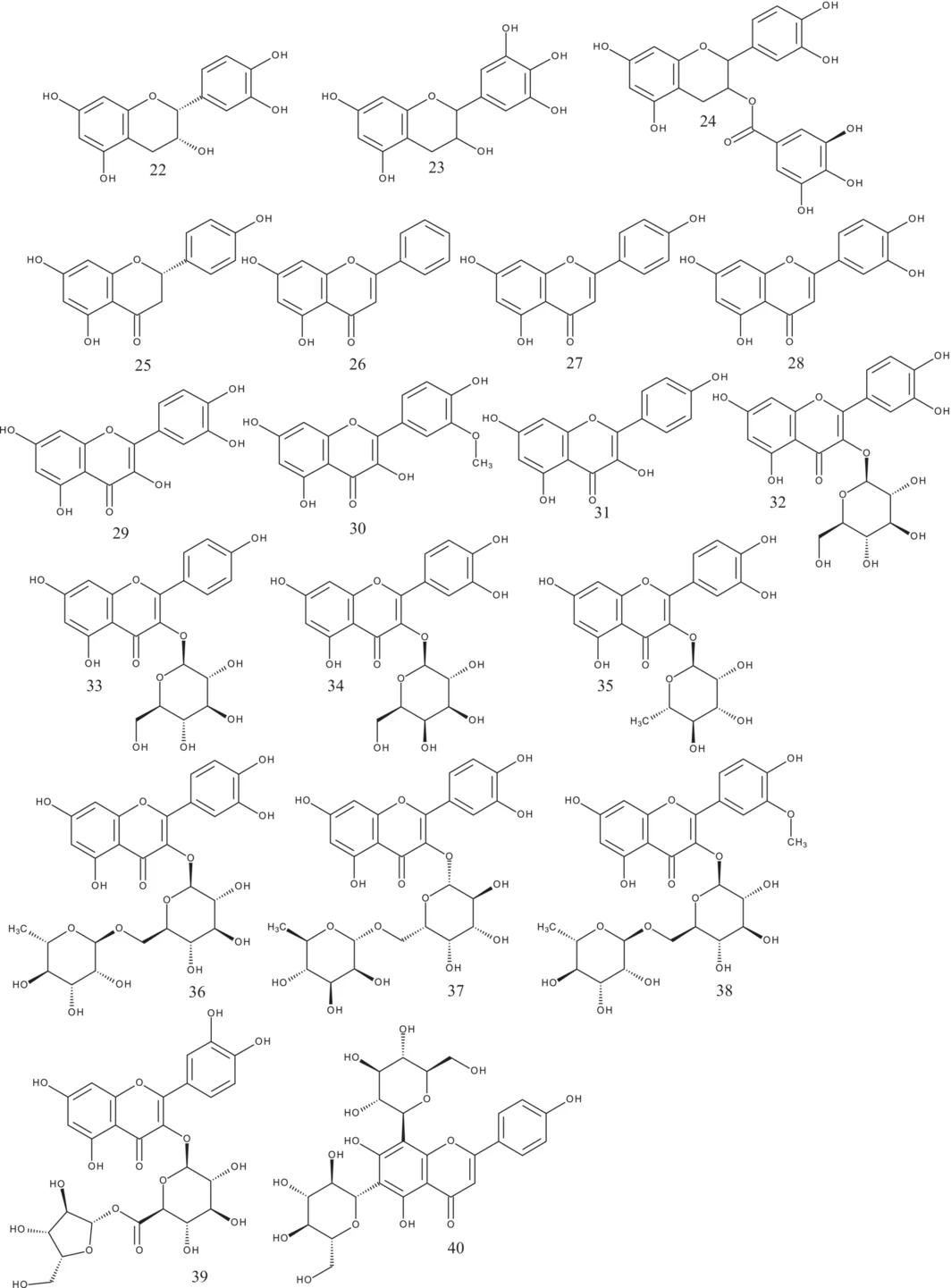
Flavonoids *R. graveolens* L. This figure illustrates the structural diversity of flavonoids and related derivatives, glycosides established for the species, demonstrating the diversity of chemical skeletons.

The flavonol glycoside rutin **36**, which was identified in samples from at least eight countries in a large study, owes its name to the genus *Ruta*, reflecting its historical isolation from this species. This is a marker compound that is not only chemotaxonomic for the genus but also its pharmacological marker (Asgharian et al. 2020; Jash et al. 2024). Flavan‐3‐ols, such as epicatechin **22**, gallocatechin **23**, and epicatechin gallate **24**, were also found in the aerial parts of the plant in samples from Portugal and Saudi Arabia. The only flavanone detected in *R. graveolens* was naringenin **25**, identified in extracts of the aerial parts of the plant collected in Algeria (Mokhtar et al. 2022).

No data are available on the chemical composition of phenolic compounds in the underground roots.

### 6.3 Coumarins

For *R. graveolens* raw materials, the presence of about 35 coumarins of various chemical structures has been established—furocoumarins, dihydrofurocoumarins, hydroxycoumarins, pyranocoumarins, as well as rare chromene derivatives, which are presented in Figure 6a,b (Table 2).

**FIGURE 6:**
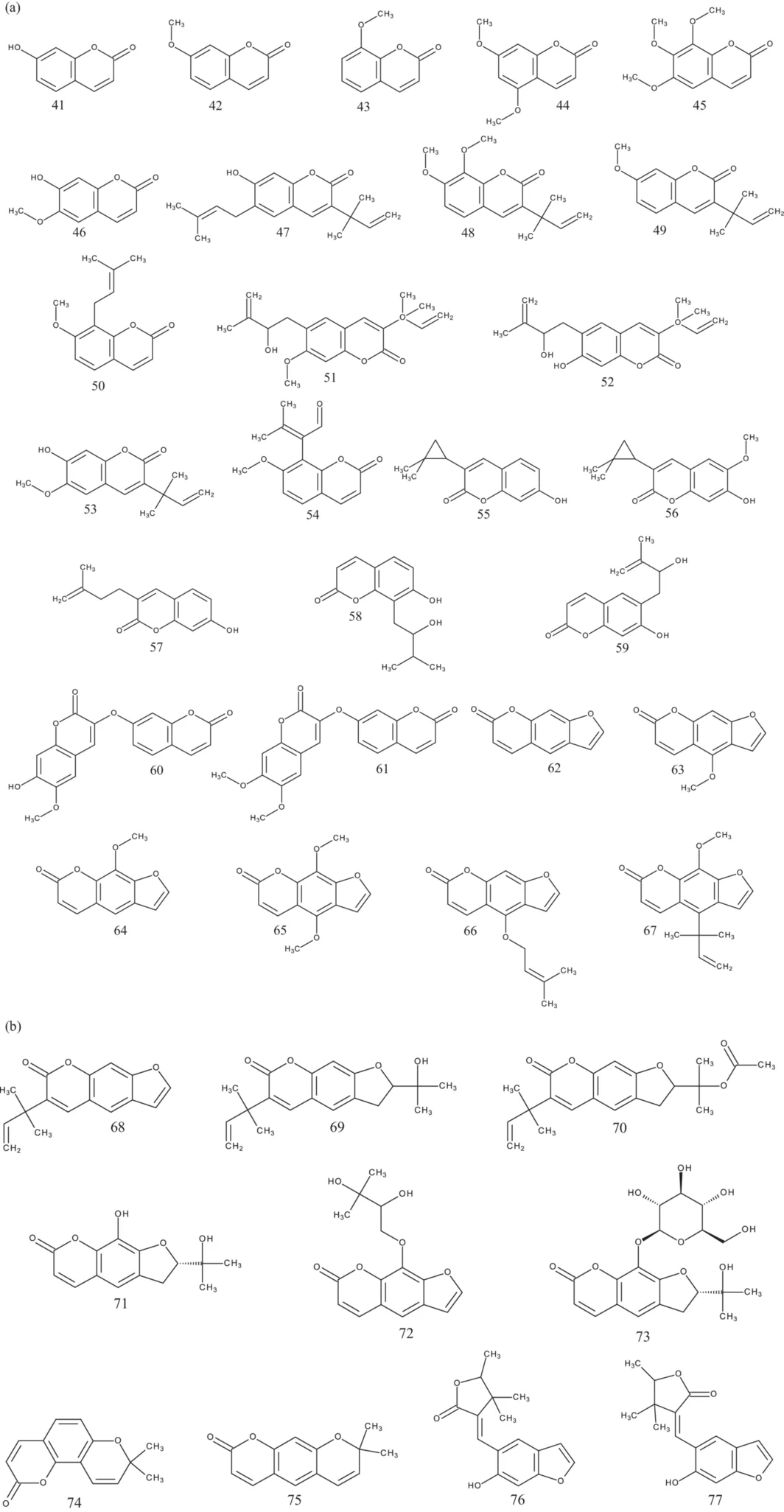
(a) Coumarins *R. graveolens* L. This figure illustrates the structural diversity of coumarins and related derivatives, including simple coumarins and furocoumarins established for the species, demonstrating the diversity of chemical skeletons. (b) Coumarins *R. graveolens* L. This figure illustrates the structural diversity of coumarins and related derivatives, including dihydrofcrocoumarins and pyranocoumarins established for the species, demonstrating the diversity of chemical skeletons.

Among simple coumarins and their derivatives, the most common are umbelliferone **41**, 7‐methoxycoumarin **42**, 5,7‐dimethoxycoumarin **44**, 6,7,8‐trimethoxycoumarin **45**, scopoletin **46**. They were isolated from the aerial parts, leaves, shoots, and roots. It should be noted that the study of lyophilized extracts from the aerial parts of *R. graveolens* using HPLC allowed the first identification of a substance from this group—8‐methoxy‐chromen‐2‐one **43** (Sahu et al. 2015). Furthermore, recent phytochemical investigations of *R. graveolens* from China led to the identification of two pairs of coumarin enantiomers: rutacycoumarin A **55** and rutacycoumarin B **56** (Feng et al. 2026).

In the leaves, roots, aerial parts, as well as in the culture of shoots in vitro, furocoumarins such as psoralen **62**, bergapten **63**, xanthotoxin **64**, isopimpinelin **65** and others were isolated, as well as dihydrofuranocoumarins—chalepin **69**, rutamarin **70**, rutaretin **71**, heraclenol **72**, rutarin **73**. Among the pyranocoumarins and their dihydroforms, only two substances were identified—seselin **74**, xanthyletin **75**.

Authors from Japan were the first to isolate 3‐(1,1‐dimethylaUyl)‐6‐(2‐hydroxy‐3‐methyl‐3‐buten‐l‐yl)‐7‐methoxycoumarin **51** and 3‐(14‐dimethylaUyl)‐6‐(2‐hydroxy‐3‐methyl‐3‐buten‐l‐yl)‐7‐hydroxycoumarin **52** by column chromatography, which are not described by other authors (Nakano et al. 2016). Additionally, three new 3′‐methyl‐3′‐butenyl coumarins were characterized: the achiral graveoumarin А **57** and two pairs of enantiomers, graveoumarin B **58** and graveoumarin C **59** (Wu et al. 2026).

A separate group of new natural compounds consists of two pairs of benzofuran enantiomers, rutabenzofuran A **76** and rutabenzofuran B **77**, recently identified for the first time in aqueous extracts of the aerial part of *R. graveolens* (Liu et al. 2023).

### 6.4 Terpenes

In addition to phenolic compounds and flavonoids, *R. graveolens* is a source of terpene compounds, represented by both monoterpenes and sesquiterpenes, which are shown in Figure 7, distinguishing this plant from others. Analysis of essential oils and extracts obtained from the aerial parts of the plant collected in various regions, such as Romania, Tunisia, Algeria, China, Peru, and Mexico, using mainly the GC–MS method, allowed the identification of more than 40 terpene compounds (Table 2).

**FIGURE 7:**
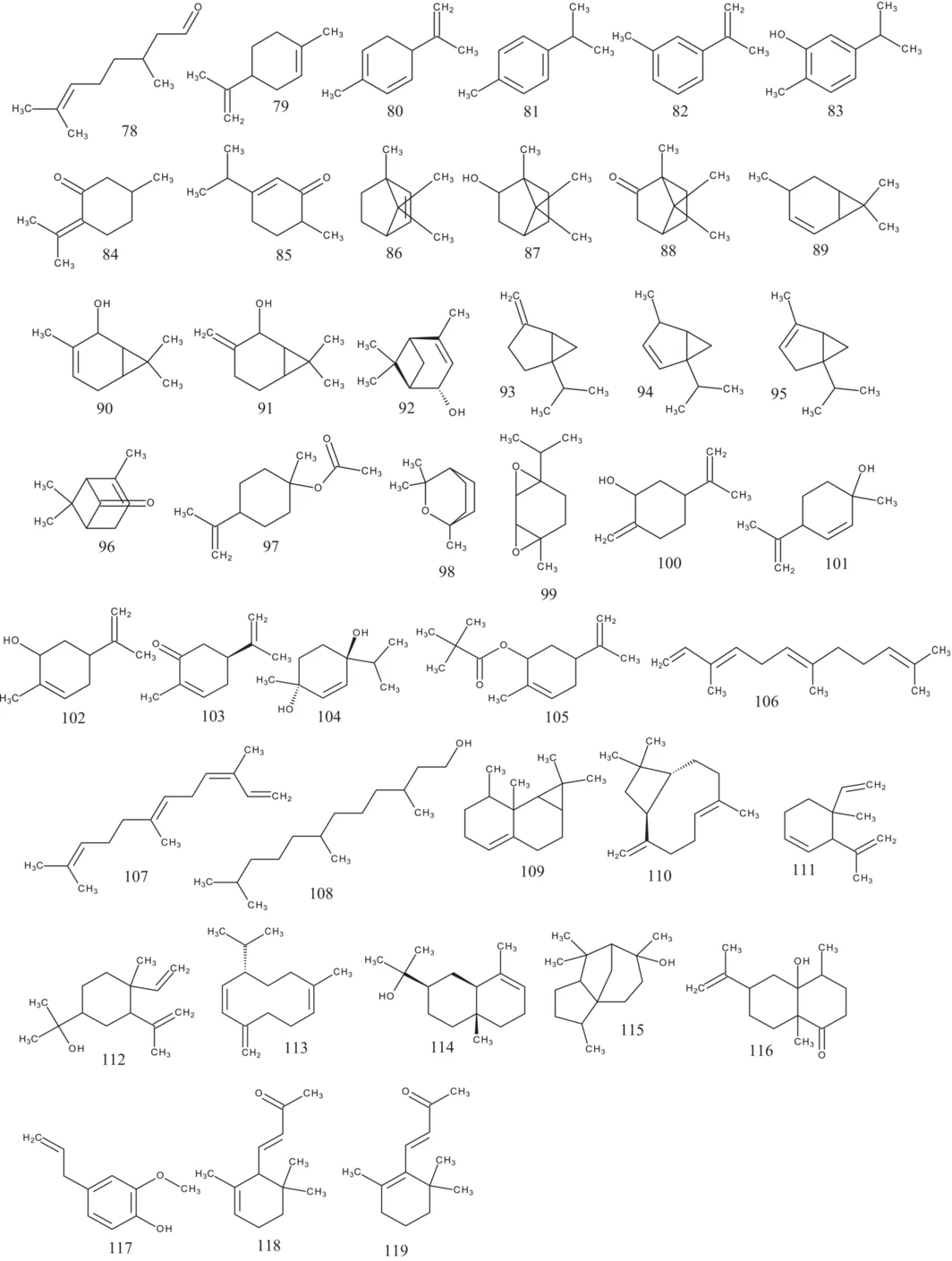
Terpenes compounds of *R. graveolens* L. This figure illustrates the structural diversity of terpene compounds and related derivatives, including monoterpenes and sesquiterpenes established for the species, demonstrating the diversity of chemical skeletons.

Both acyclic and bicyclic compounds were found, among the monoterpenes: citronellal **78**, α‐limonene **79**, p‐mentha‐1,5,8‐triene **80**, p‐cymene **81**, m‐cymenene **82** and others. Sesquiterpenes include α‐farnesene **106**, (Z,E)‐α‐farnesene **107**, hexa‐hydro‐farnesol **108**, calarene **109**, β‐caryophyllene **110**, geijerene **111**, elemol **112** and others.

Using GC‐q‐TOF‐MS, a number of new terpene compounds were found in *R. graveolens* raw materials from Mexico, not in essential oil, but in methanol extracts from the aerial parts and roots of *R. graveolens* , indicating a wider chemical diversity of the plant than previously thought. Among the identified compounds were: citronellal **78**, p‐mentha‐1,5,8‐triene **80**, m‐cymenene **82**, pulegone **84**, carvenone **85**, and others (Reyes‐Vaquero et al. 2024).

*R. graveolens* has a diverse terpene composition, with most of the identified compounds being natural components of essential oil, and some obtained from extracts. This also indicates the plant's significant pharmacological potential.

### 6.5 Alkaloids

Alkaloids are the second largest group of substances found in *R. graveolens* . As a result of research, a number of furoquinolone, quinolone, acridone, and acridoinoquinoline alkaloids were isolated from *R. graveolens* raw materials, as shown in Figure 8a,b. In extracts of the above‐ground parts of the plant collected in various geographical regions (China, Saudi Arabia, Egypt, Japan, Brazil, Mexico, European countries), more than 40 alkaloids belonging to different structural classes were identified (Table 2).

**FIGURE 8:**
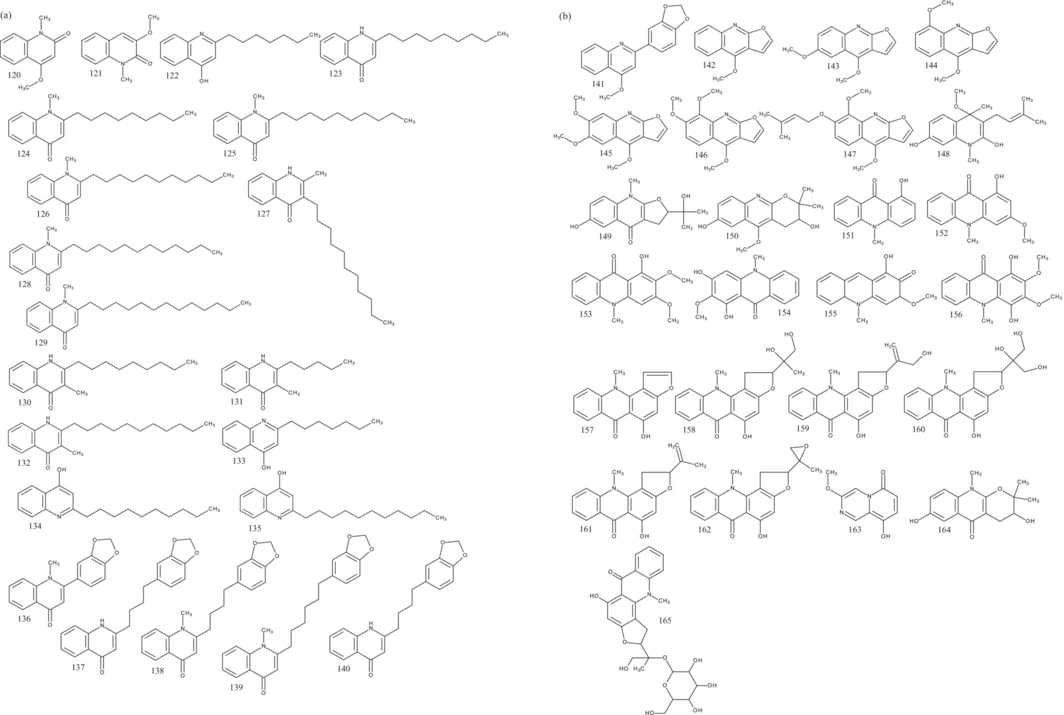
(a) Alkaloids of *R. graveolens* L. This figure illustrates the structural diversity of alkaloids and related derivatives, including quinoline derivatives, established for the species, demonstrating the diversity of chemical skeletons. (b) Alkaloids of *R. graveolens* L. This figure illustrates the structural diversity of alkaloids and related derivatives, including furoquinoline, acridone derivatives, established for the species, demonstrating the diversity of chemical skeletons.

Quinolone alkaloids isolated from the aerial parts of the plant and roots are represented by compounds such as N‐methyl‐4‐methoxy‐2‐quinolone **120**, 3‐methoxy‐l‐methyl‐2‐qunolinone **121**, 2‐heptyl‐4(1H)quinolone **122**, 2‐nonyl‐4(lH)‐quinolone **123**, 1‐methyl‐2‐nonyl‐4(1H)‐ quinolone **124** and others. These alkaloids were detected using UPLC‐QTOF‐MS, CC, HPLC, which allowed the identification of both known and new structures. Among the furoquinoline alkaloids identified in *R. graveolens* raw materials are dictamnine **142**, pteleine **143**, γ‐fagarine **144**, kokusaginine **145**, skimmianine **146** and others. These compounds were found in various parts of the plant, including leaves, stems, fruits, and tissues cultivated in vitro.

Acridone alkaloids isolated from leaves and roots include 1‐hydroxy‐10‐methylacridone **151**, 1‐hydroxy‐3‐methoxy‐10‐methyl‐9(10H)‐acridinone **152**, arborinine **153**, 1,3‐dihydroxy‐2‐methoxy‐10‐methyl‐9‐acridone **154**, 1‐hydroxy‐3‐methoxy‐N‐methylacridone **155**, and others.

Research from Egypt reports on a new chemical structure – (4S)‐1,4‐dihydro‐4‐methoxy‐1,4‐dimethyl‐3‐(3‐methylbut‐2‐enyl)quinoline‐2,7‐diol **148**, which was first isolated by column chromatography from plant leaves (Salib et al. 2014). According to the article, authors from China isolated another new compound, rutagrarin A **150**, and also isolated the pyrazine alkaloid xylogranatinin **163** from the aerial parts of the plant, adding another group of biologically active substances to the chemical profile of this plant (Ainiwaer et al. 2023).

Unlike other classes of substances, alkaloids were mainly isolated using column chromatography, which allows new or poorly studied compounds to be obtained.

Thus, *R. graveolens* is a rich source of bioactive components, which are notable for their significant diversity in structure and compound groups. The generalization of data on individual groups of metabolites allows us to deepen our understanding of the diversity of chemical composition, trace the influence of geographical origin on the variability of composition, comprehensively assess the chemical potential of the plant, and create a basis for further research into its biological activity in the pharmaceutical and medical fields. In particular, within the scope of this study, 38 compounds have been identified as specific markers for *R. graveolens* and related species of the Rutaceae family; these compounds are highlighted with asterisks (*) in the respective tables.

## 7 Pharmacological Activity of *R. graveolens*

Available experimental studies confirm the broad pharmacological potential of *R. graveolens* , including antimicrobial, antifungal, antiviral, anti‐inflammatory, neuroprotective, anti‐asthmatic, antitumor, anticholinesterase, and other types of activity (Forouhar et al. 2019). This spectrum of action is due to the presence of various classes of bioactive compounds in the plant, namely alkaloids, flavonoids, coumarins, terpenoids, and others.

Despite numerous publications, authors mostly focus on specific areas (Colucci‐D'Amato and Cimaglia 2020; Pandey et al. 2016; Pushpa et al. 2015), leaving out a holistic view of the multifaceted effects of *R. graveolens* . The generalization of the results allows not only to confirm the broad therapeutic potential of this plant but also to identify gaps in current research, which justifies and determines the need for promising targeted directions for further preclinical and clinical studies.

### 7.1 Antibacterial Activity

Modern research confirms the significant potential of *R. graveolens* extracts and essential oil. A promising direction is the comparison of the activity of different extracts and the identification of key bioactive components that determine the effectiveness against gram‐positive and gram‐negative pathogens (Table 3).

**TABLE 3:** Antimicrobial activity of *R. graveolens* .

Strain	Extract	Plant organs	MIC, mg/mL	MBC, mg/mL	ZOI, mm	References
Gram‐positive						
*Bacillus cereus*	Methanolic	Leaf	0.37	0.75	—	Elansary et al. 2020
	Methanolic	Aerial parts	12.50	100.00	—	Pavlovic et al. 2014
	Ethanolic	Aerial parts	6.25	25.00	—	Pavlovic et al. 2014
	Essential oil	Aerial parts	0.075	< 100	14.50	Jeon et al. 2014
	Essential oil	Leaf	0.001	—	25.60	França Orlanda and Nascimento 2015
	Essential oil	Aerial parts	1.00		26.60	Reddy et al. 2016
*Bacillus subtilis*	Methanolic	Aerial parts	—	—	~16.00	Pushpa et al. 2015
	Ethanolic	Leaf	—	—	17.90	Pandey et al. 2016
	Water	Aerial parts	—	—	100.00	Hashemi Karouei 2015
	Ethanolic	Aerial parts	—	—	26.00	Hashemi Karouei 2015
	Methanolic	Aerial parts	—	—	22.00	Hashemi Karouei 2015
	Chloroform	Fresh leaves	—	—	18.00	Amabye 2015
	Methanolic	Fresh leaves	—	—	17.00	Amabye 2015
	Ethanolic	Fresh leaves	—	—	15.00	Amabye 2015
	Water	Fresh leaves	—	—	—	Amabye 2015
	NADES	Dried leaves	0.125	—	—	Pavić et al. 2019
*Enterococcus faecalis*	Ethanolic	Stem	100.00	—	3.20	Al‐Muffti et al. 2021
	Ethanolic	Leaf	100.00	—	4.50	Al‐Muffti et al. 2021
	Essential oil	Aerial parts	0.001	—	27.10	Reddy et al. 2016
*Listeria monocytogenes*	Ethanolic	Aerial parts	1.25	—	11.75	Mokhtar et al. 2022
	Methanolic	Leaf	0.43	0.89	—	Elansary et al. 2020
	Methanolic	Aerial parts	12.50	100	—	Pavlovic et al. 2014
	Ethanolic	Aerial parts	6.25	6.25	—	Pavlovic et al. 2014
	Essential oil	Aerial parts	0.075	0.10	13.0	Jeon et al. 2014
	Essential oil	Aerial parts	0.0007	—	18.30	Reddy et al. 2016
*Micrococcus flavus*	Methanolic	Leaf	0.49	0.99	—	Elansary et al. 2020
	Essential oil	Leaf	0.00075	—	19.00	França Orlanda and Nascimento 2015
	Essential oil	Aerial parts	0.00148	—	21.20	Reddy et al. 2016
*Micrococcus luteus*	Essential oil	Leaf	0.00089	—	17.00	Pushpa et al. 2015
	Essential oil	Aerial parts	0.001	—	19.06	Reddy et al. 2016
*Staphylococcus aureus*	Polyphenol	Aerial parts	0.625	—	14.37	Mokhtar et al. 2022
	Methanolic	in vitro biomass	0.50	—	—	Szewczyk et al. 2022
	Methanolic	Aerial parts	—	—	~25	Pushpa et al. 2015
	Methanolic	Leaf	0.31	0.84	—	Elansary et al. 2020
	Ethanolic	Stem	—	—	10.00	Al‐Muffti et al. 2021
	Ethanolic	Leaf	—	—	~4.70	Al‐Muffti et al. 2021
	Ethanolic	Leaf	—	—	17.00	Pandey et al. 2016
	Ethyl acetate	Fresh roots	—	—	2.00	Al‐Sokari and El Sheikha 2015
	Chloroformic	Fresh leaves	—	—	14.00	Amabye 2015
	Methanolic	Fresh leaves	—	—	13.00	Amabye 2015
	Ethanolic	Fresh leaves	—	—	15.00	Amabye 2015
	Water	Fresh leaves	—	—	—	Amabye 2015
	Methanolic	Leaf	—	—	12.00	Kumar et al. 2014
	Chloroformic	Leaf	—	—	6.00	Kumar et al. 2014
	Ethyl acetate	Leaf	—	—	5.00	Kumar et al. 2014
	Methanolic	Aerial parts	25.00	> 100	—	Pavlovic et al. 2014
	Ethanolic	Aerial parts	12.50	25.00	—	Pavlovic et al. 2014
	Water	Leaf	—	—	~7.60	Sheykhani et al. 2020
	Methanolic	Leaf	—	—	~7.20	Sheykhani et al. 2020
	Water	Aerial parts	—	—	17.00	Hashemi Karouei 2015
	NADES	Dried leaves	0.125	—	—	Pavić et al. 2019
	Ethanolic	Aerial parts	—	—	22.00	Hashemi Karouei 2015
	Methanolic	Aerial parts	—	—	22.00	Hashemi Karouei 2015
	Essential oil	Leaf	0.001	—	22.00	França Orlanda and Nascimento 2015
	Essential oil	Aerial parts	0.001	—	23.40	Reddy et al. 2016
Methicillin‐resistant *Staphylococcus aureus*	Ethanolic	Aerial parts	0.78	—	From 26.00 to 30.00	Rezk et al. 2022
*Staphylococcus intermedius*	Essential oil	Aerial parts	0.025	0.05	14.30	Jeon et al. 2014
*Staphylococcus epidermidis*	EtoAc	Fresh roots	—	—	1.00	Al‐Sokari and El Sheikha 2015
*Streptococcus pyogenes*	Ethanolic	Stem	—	—	19.50	Al‐Muffti et al. 2021
	Ethanolic	Leaf	—	—	9.00	Al‐Muffti et al. 2021
*Streptococcus mitis*	Methanolic	Aerial parts	—	—	~12.00	Pushpa et al. 2015
*Streptococcus mutans*	Methanolic	Aerial parts	—	—	12.25	Salman et al. 2018
*Streptococcus sobrinus*	Methanolic	Aerial parts	—	—	12.67	Salman et al. 2018
Gram‐negative						
*Acinetobacter baumannii*	Essential oil	Aerial parts	0.00122	—	18.60	Reddy et al. 2016
*Citrobacter freundii*	Essential oil	Aerial parts	0.001	—	16.02	Reddy et al. 2016
*Escherichia coli*	Polyphenol	Aerial parts	1.25	—	10.25	Mokhtar et al. 2022
	Methanolic	Aerial parts	—	—	~15.00	Pushpa et al. 2015
	Methanolic	Leaf	0.36	0.96		Elansary et al. 2020
	Ethanolic	Leaf	—	—	15.30	Al‐Muffti et al. 2021
	Ethanolic	Stem	—	—	~6.00	Al‐Muffti et al. 2021
	Ethanolic	Leaf	—	—	19.00	Pandey et al. 2016
	EtoAc	Fresh roots	—	—	8.00	Al‐Sokari and El Sheikha 2015
	Chloroformic	Fresh leaves	—	—	24.00	Amabye 2015
	Methanolic	Fresh leaves	—	—	17.00	Amabye 2015
	Ethanolic	Fresh leaves	—	—	19.00	Amabye 2015
	Water	Fresh leaves	—	—	—	Amabye 2015
	Methanolic	Leaf	—	—	6.00	Kumar et al. 2014
	Ethyl acetate	Leaf	—	—	6.00	Kumar et al. 2014
	Petroleum ether	Leaf	—	—	6.00	Kumar et al. 2014
	Methanolic	Aerial parts	100.00	> 100	—	Pavlovic et al. 2014
	Ethanolic	Aerial parts	12.50	> 100	—	Pavlovic et al. 2014
	Water	Leaf	—	—	~7.60	Sheykhani et al. 2020
	Methanolic	Leaf	—	—	~7.20	Sheykhani et al. 2020
	Water	Aerial parts	—	—	15.00	Hashemi Karouei 2015
	Ethanolic	Aerial parts	—	—	20.00	Hashemi Karouei 2015
	Methanolic	Aerial parts	—	—	—	Hashemi Karouei 2015
	NADES	Dried leaves	0.125	—	—	Pavić et al. 2019
	Essential oil	Leaf	0.001	—	17.70	França Orlanda and Nascimento 2015
	Essential oil	Aerial parts	0.00065	—	17.70	Reddy et al. 2016
*Enterobacter aerogenes*	Essential oil	Leaf	0.0014	—	12.54	França Orlanda and Nascimento 2015
	Essential oil	Aerial parts	0.072	—	12.57	Reddy et al. 2016
*Enterobacter cloacae*	Essential oil	Aerial parts	0.00089	—	18.00	Reddy et al. 2016
*Klebsiella pneumoniae*	Methanolic	Aerial parts	—	—	~37.00	Pushpa et al. 2015
	Ethanolic	Leaf	—	—	15.00	Pandey et al. 2016
	EtoAc	Fresh roots	—	—	3.00	Al‐Sokari and El Sheikha 2015
	Methanolic	Leaf	—	—	12.00	Kumar et al. 2014
	Chloroformic	Leaf	—	—	6.00	Kumar et al. 2014
	Ethyl acetate	Leaf	—	—	6.00	Kumar et al. 2014
	Essential oil	Aerial parts	0.00158	—	18.10	Reddy et al. 2016
*Legionella pneumophila* (all serogroups)	Essential oil	Aerial parts	0.02–0.40	—	—	Chaftar et al. 2015
*Pseudomonas aeruginosa*	Polyphenol	Aerial parts	2.50	—	9.00	Mokhtar et al. 2022
	Methanolic	Leaf	0.39	0.98		Elansary et al. 2020
	Ethanolic	Leaf	—	—	~7.00	Al‐Muffti et al. 2021
	Ethanolic	Stem	—	—	~4.80	Al‐Muffti et al. 2021
	Ethanolic	Leaf	—	—	12.40	Pandey et al. 2016
	EtoAc	Fresh roots	—	—	7.00	Al‐Sokari and El Sheikha 2015
	Chloroformic	Fresh leaves	—	—	8.00	Amabye 2015
	Methanolic	Fresh leaves	—	—	11.00	Amabye 2015
	Ethanolic	Fresh leaves	—	—	16.00	Amabye 2015
	Water	Fresh leaves	—	—	—	Amabye 2015
	Methanolic	Aerial parts	12.50	25.00	—	Pavlovic et al. 2014
	Ethanolic	Aerial parts	6.25	> 100	—	Pavlovic et al. 2014
	Water	Aerial parts	—	—	12.00	Hashemi Karouei 2015
	Ethanolic	Aerial parts	—	—	—	Hashemi Karouei 2015
	NADES	Dried leaves	0.0625	—	—	Pavić et al. 2019
	Methanolic	Aerial parts	—	—	20.00	Hashemi Karouei 2015
	Essential oil	Leaf	0.075	—	8.30	França Orlanda and Nascimento 2015
	Essential oil	Aerial parts	0.001	—	15.30	Reddy et al. 2016
*Proteus mirabilis*	EtoAc	Fresh roots	—	—	5.00	Al‐Sokari and El Sheikha 2015
	Essential oil	Aerial parts	0.00076	—	22.00	Reddy et al. 2016
*Salmonella enterica*	Essential oil	Aerial parts	0.025	0.075	21.50	Jeon et al. 2014
*Salmonella enteritidis*	Methanolic	Aerial parts	> 100	> 100	—	Pavlovic et al. 2014
	Ethanolic	Aerial parts	50.00	> 100	—	Pavlovic et al. 2014
*Salmonella typhimurium*	Polyphenol	Aerial parts	2.50	—	9.37	Mokhtar et al. 2022
	Ethanolic	Leaf	—	—	16.70	Pandey et al. 2016
	Essential oil	Aerial parts	0.025	0.05	15.20	Jeon et al. 2014
	Essential oil	Aerial parts	0.00134	—	13.03	Reddy et al. 2016
*Salmonella typhi*	Methanolic	Aerial parts	—	—	~25.00	Pushpa et al. 2015
	Essential oil	Leaf	0.001	—	12.40	França Orlanda and Nascimento 2015
*Shigella sonnei*	Essential oil	Aerial parts	0.025	0.05	14.10	Jeon et al. 2014

Abbreviations: MBC, Minimum Bactericidal Concentration; MIC, Minimum Inhibitory Concentration; ZOI, Zone of Inhibition.

The antibacterial activity of *R. graveolens* extracts and essential oils has been confirmed by numerous studies. In general, it has been established that *R. graveolens* has a more pronounced effect against Gram‐positive bacteria (*Staphylococcus aureus, Bacillus cereus, Listeria monocytogenes, Streptococcus* spp.) compared to Gram‐negative bacteria (*Escherichia coli, Pseudomonas aeruginosa, Klebsiella pneumoniae, Salmonella* spp.), which are characterized by higher natural resistance (Al‐Muffti et al. 2021; Amabye 2015; Kumar et al. 2014; Hashemi Karouei 2015; Mokhtar et al. 2022; França Orlanda and Nascimento 2015; Pandey et al. 2016; Pavlovic et al. 2014; Pushpa et al. 2015; Al‐Sokari and El Sheikha 2015; Sheykhani et al. 2020; Jazzar et al. 2023).

Methanol, ethanol, chloroform, and polyphenolic extracts showed a pronounced inhibitory effect, with efficacy depending on the type of solvent and plant organ. Ethanol extracts most often showed a stronger effect against *S. aureus* and *Bacillus* spp. (Al‐Muffti et al. 2021; Amabye 2015; Hashemi Karouei 2015; Pavlovic et al. 2014), while methanol and chloroform extracts exceeded their activity against *E. coli* and *Klebsiella* spp. in a number of studies (Amabye 2015; Elansary et al. 2020; Kumar et al. 2014; Pushpa et al. 2015). A separate study demonstrated the absence of action of ethanol extract against *S. aureus* and *E. coli* (Jabir et al. 2022). Notably, aqueous extracts often show no activity against common pathogens (Amabye 2015; Kumar et al. 2014; Saeidinia et al. 2016). This phenomenon is primarily explained by the low solubility of key antibacterial metabolites of *R. graveolens* , in particular acridone alkaloids, furanocoumarins, and terpenoids in water. These compounds are predominantly lipophilic (hydrophobic) and do not dissolve effectively in polar aqueous media. Phytochemical screening confirms that aqueous extracts are limited to polar steroids and phenolic compounds with lower antibacterial potential (Amabye 2015). Regarding the role of solvents, it is clarified that ethanol and methanol are not the only cause of the observed activity, since standard experimental protocols included evaporation of solvents to obtain a dry residue (Kumar et al. 2014) or drying the discs for 48 h before testing (Saeidinia et al. 2016), which eliminates the direct antiseptic effect of the alcohol itself. This confirms that the therapeutic effect comes precisely from the extracted plant metabolites. The rare reports of aqueous activity can be explained by variations in extraction conditions, such as the use of boiling, which allows for a higher concentration of polar phenolic acids (Sheykhani et al. 2020).

The study demonstrates the efficacy of extracts obtained using innovative natural deep eutectic solvents (NADES) based on choline chloride and citric acid. These extracts were found to have pronounced activity against a wide range of pathogens, including *S. aureus, B. subtilis, E. coli*, and especially *P. aeruginosa* (MIC = 0.0625 mg/mL). Statistical analysis revealed a very strong correlation between the total phenolic content and antibacterial efficacy, while the rutin content did not significantly affect bacterial inhibition (Pavić et al. 2019).

*R. graveolens* essential oil consistently demonstrated high activity, especially against Gram‐positive bacteria: *S. aureus, B. cereus, L. monocytogenes, Enterococcus faecalis*, with inhibition zones of over 20 mm and low MIC values (0.7–1.0 μg/mL) (França Orlanda and Nascimento 2015; Reddy et al. 2016). The antibacterial properties are associated with a high content of polyphenols (chlorogenic acid, p‐coumaric acid, quercetin) and alkaloids (γ‐phagarine, cocusagin), as well as specific components of essential oil, in particular undecan‐2‐one and 2‐isopropyl‐5‐methylphenol (Chaftar et al. 2015; Jeon et al. 2014; Salman et al. 2018).

The effect of *R. graveolens* on resistant pathogens is interesting: it has been shown to have an antibacterial effect against Methicillin‐resistant Staphilococcus (MRSA) by suppressing the expression of the mecA, icaA, and icaD genes (Rezk et al. 2022), as well as strong anti‐legionellosis activity of essential oil (MIC ≤ 0.06 mg/mL) (Chaftar et al. 2015).

The data presented indicate that *R. graveolens* is a promising source of bioactive compounds with a broad spectrum of antibacterial activity, with efficacy depending significantly on the type of extract, plant organ, pathogen studied, and experimental conditions.

### 7.2 Antifungal Activity

In addition to its antibacterial action, research into the antifungal activity of *R. graveolens* is of considerable interest. This is due to the prevalence of fungal infections and the growing resistance of pathogens to traditional drugs, which requires the search for new sources of effective compounds. Scientific literature contains data on the activity of *R. graveolens* extracts and essential oil against a wide range of pathogenic fungi. Particular attention is paid to representatives of the genera *Penicillium, Candida*, and *Aspergillus*, which are among the most clinically significant causative agents of mycoses in humans (Table 4).

**TABLE 4:** Antifungal activity of *R. graveolens* .

Strain	Extract	Plant organs	MIC, mg/mL	MFC, mg/mL	ZOI, mm	References
*Alternaria sps*.	Methanolic	Aerial parts	—	—	10.00	Pushpa et al. 2015
*Alternaria alternata*	Essential oils	Aerial parts	0.0098	—	22.30	Reddy et al. 2016
*Aspergillus flavus*	Methanolic	Leaf	0.33	0.84	—	Elansary et al. 2020
	Essential oils	Aerial parts	0.00905	—	25.60	Reddy et al. 2016
*Aspergillus fumigates*	Essential oils	Aerial parts	0.0045	—	14.30	Reddy et al. 2016
*Aspergillus niger*	Methanolic	Aerial parts	—	—	13.00	Pushpa et al. 2015
	Methanolic	Leaf	0.61	1.12	—	Elansary et al. 2020
	Ethanolic	Aerial parts	25.00	—	—	Pavlovic et al. 2014
*Aspergillus ochraceus*	Methanolic	Leaf	0.47	1.02	—	Elansary et al. 2020
*Candida albicans*	Methanolic	Aerial parts	—	—	16.00	Pushpa et al. 2015
	Methanolic	Leaf	0.78	1.77	—	Elansary et al. 2020
	Ethanolic	Aerial parts	25.00	—	—	Pavlovic et al. 2014
	EtoAc extract	Root	—	—	1.00	Al‐Sokari and El Sheikha 2015
	Essential oils	Aerial parts	0.022	—	35.10	Reddy et al. 2016
*Cryptococcus sps*.	Methanolic	Aerial parts	—	—	13.00	Pushpa et al. 2015
*Fusarium oxyporum*	Essential oils	Aerial parts	0.00906	—	20.02	Reddy et al. 2016
*Microsporium canis*	EtoAc	Root	—	—	6.00	Al‐Sokari and El Sheikha 2015
*Penicillium funiculosum*	Methanolic	Leaf	0.53	1.05	—	Elansary et al. 2020
*Penicillium ochrochloron*	Methanolic	Leaf	0.64	1.54	—	Elansary et al. 2020
*Penicillium* spp.	Methanolic	Aerial parts	—	—	17.00	Pushpa et al. 2015
*Trichoderma sps*.	Methanolic	Aerial parts	—	—	17.00	Pushpa et al. 2015

Abbreviations: MFC, Minimum Fungicidal Concentration; MIC, Minimum Inhibitory Concentration; ZOI, Zone of Inhibition.

The antifungal activity of *R. graveolens* has been confirmed in a number of studies. The methanol extract was effective against six fungal isolates, with *Penicillium* spp. and *Trichoderma* spp. being the most sensitive, as well as *Aspergillus niger*, *Candida albicans* , and *Cryptococcus* spp. Interestingly, the activity of the extract against *Cryptococcus* sps. was higher than that of the standard nystatin preparation (Pushpa et al. 2015).

In a study by Serbian authors, methanol and ethanol extracts of cultivated and wild *R. graveolens* were compared. The most pronounced activity was observed in the ethanol extract of wild plants at the beginning of flowering against *C. albicans* and *A. niger* (MIC = MBC 25 mg/mL) (Pavlovic et al. 2014).

Studies have also confirmed the activity of root extract against *Microsporium canis* (inhibition zone 6.0 mm) and moderate activity against *C. albicans* (Al‐Sokari and El Sheikha 2015). The methanol extract of the leaves showed moderate antifungal activity against most of the isolates studied, with the most pronounced activity against *Aspergillus flavus* and *A. ochraceus* . Phytochemical analysis revealed the presence of polyphenols (quercetin, chlorogenic, p‐coumaric, protocatechuic, and caffeic acids, rutoside), which are likely responsible for the antifungal activity (Elansary et al. 2020).

The most significant results were demonstrated by *R. graveolens* essential oil, which showed maximum activity against *C. albicans* (inhibition zone 35.10 mm; MIC 22.00 μg/mL). At the same time, *A. fumigatus* was the most sensitive strain in terms of MIC (4.50 μg/mL), while *C. herbarum* , *A. alternata* , *A. flavus* , and *F. oxysporum* showed moderate sensitivity (Reddy et al. 2016).

Thus, essential oil and extracts of *R. graveolens* exhibit significant antibacterial activity mainly against gram‐positive bacteria, as well as antifungal activity against *Candida, Aspergillus*, and other pathogenic fungi. Methanol and ethanol extracts were found to be the most active, while essential oil was characterized by the lowest MIC values. Biological activity is associated with the presence of polyphenols, flavonoids, alkaloids, and specific volatile components.

### 7.3 Antiviral Activity

It has been established that the aqueous extract of *R. graveolens* exhibits antiviral activity against SARS‐CoV‐2. In vitro studies have shown its potential inhibitory properties with an IC_50_ value of 1.299 mg/mL and a cytotoxic concentration CC_50_ of 11.991 mg/mL. In addition, *in silico* analysis identified four active compounds—amentoflavone, agathisflavone, vitamin P, and daphnetin—that bind to the S‐protein of the virus. Specifically, the mechanism of action involves disrupting the molecular interaction between the viral spike glycoprotein and the host ACE2 receptor, thereby impeding membrane fusion and initiating viral entry. Experimental plaque reduction assays demonstrated a dose‐dependent inhibitory effect; for instance, the extract doses of 1.50 and 0.75 μg/μL effectively inhibited SARS‐CoV‐2 proliferation by 77% and 68%, respectively. This suggests a possible mechanism of action and identifies them as promising candidates for therapeutic intervention in complications associated with SARS‐CoV‐2 (Khandoker Minu et al. 2024).

It was found that the aqueous‐alcoholic extract of *R. graveolens* exhibits pronounced antiviral activity against acyclovir‐resistant herpes simplex virus type 1 (HSV‐1). In vitro studies on HeLa cells showed its significant inhibitory potential with an IC50 of 75 μg/mL and a cytotoxicity concentration CC50 of 1150 μg/mL, which provides a high selectivity index (SI) of 15.33. The mechanism of action of the extract is to inhibit viral replication within the cell, in particular by interfering with the expression of alpha genes or the functioning of proteins of the initial phase of replication, which is confirmed by a decrease in the titer of the virus (Ebrahimi et al. 2021).

The sterol fraction isolated from the leaves of *R. graveolens* has been shown to exhibit significant antiviral activity, particularly against the avian influenza virus (H5N1). In vitro studies have shown that the sterol mixture inhibits viral replication with an IC_50_ of 7.8 μg/mL, while the cytotoxic concentration TC_50_ is 0.12 mg/mL. Gas chromatographic analysis has identified the key active components, phytosterols, dominated by β‐sitosterol, stigmasterol, and campesterol. The mechanism of action of these compounds is to directly inhibit viral replication (Abou Baker et al. 2017).

### 7.4 Effects on the Central Nervous System

*R. graveolens* is noteworthy as a source of polyphenols, flavonoids, and alkaloids, which may potentially influence key mechanisms of neurodegeneration, including oxidative stress. Recent studies have shown that extracts and essential oil of *R. graveolens* can modulate the activity of cholinesterases and monoamine oxidases, as well as exert anti‐inflammatory and antioxidant effects, which opens up prospects for their use in the prevention and treatment of neurodegenerative disorders (Table 5).

**TABLE 5:** Central nervous system effects of *R. graveolens* .

Extract	Plant organs	Result	References
Essential oils	Aerial parts	Inhibition of BChE with IC_50_ = 333.1 μg/mL; inactive against AChE (IC_50_ > 0.5 μg/mL)	Saeedi et al. 2022
Water	Leaf	The extract reduced the volume of cerebral infarction (60%) and improved neurological outcomes in a rat model of transient focal cerebral ischemia (tMCAO). The extract contained 10 mg/kg of rutin.	Campanile et al. 2022
Hydroalcoholic	Leaf	The extract at doses of 100 and 300 mg/kg and rutin significantly improved learning and spatial memory, as well as secondary latency.	Asgharian et al. 2020
Methanolic	Herb	AChE inhibition with IC_50_ = 250 μg/mL. At a concentration of 400 μg/mL, the extract inhibited AChE by 73.8% and BChE by 29.0%.	Talić et al. 2014
Water	Herb	AChE inhibition with IC_50_ = 50 μg/mL. At a concentration of 400 μg/mL, the aqueous extract inhibited AChE by 80.0% and BChE by 32.2%.	Talić et al. 2014
Dichloromethane	Aerial parts	Inhibition by the extract (concentration 9.78 mg/mL) MAO‐B—89.98%, MAO‐A—88.22%; low selectivity (SI (hMAO‐B selectivity index) = 1.020).	Kozioł et al. 2020

Abbreviations: AChE, Acetylcholinesterase; BChE, Butyrylcholinesterase; hMAO‐A, Human Monoamine Oxidase A; hMAO‐B, Human Monoamine Oxidase B; IC_50_, Half Maximal Inhibitory Concentration; SI, Selectivity Index; tMCAO, Transient Middle Cerebral Artery Occlusion.

Essential oil from the aerial parts of *R. graveolens* (Iran) showed inhibitory activity against butyrylcholinesterase (BChE) in vitro with an IC_50_ value of 333.1 μg/mL (Saeedi et al. 2022). Other authors noted the pronounced effect of *R. graveolens* extracts on acetylcholinesterase (AChE) and BChE, the inhibition of which is considered a promising strategy for the treatment of Alzheimer's disease. The aqueous extract showed the strongest activity against AChE (IC_50_ = 50 mg/mL), while for the methanol extract, the IC_50_ was 250 μg/mL. At a concentration of 400 μg/mL, the aqueous extract inhibited AChE by 80.0% and BuChE by 32.2%, which partially contradicts the data on the activity of essential oil (Talić et al. 2014).

The aqueous extract of *R. graveolens* enriched with rutin (10 mg/kg) demonstrated a neuroprotective effect in a model of transient middle cerebral artery occlusion (tMCAO) in rats. The treatment reduced infarct volume, improved neurological parameters, suppressed proinflammatory activation of astrocytes and microglia (decreased expression of C3 and iNOS in GFAP‐ and TMEM119‐positive cells), and increased BDNF mRNA levels in ventral midbrain cultures. The effect of the extract was greater than that of rutin alone, indicating a synergistic effect of its polyphenolic components (quercetin, epigallocatechin‐3‐gallate, isorhamnetin, and isorhamnetin‐3‐O‐rutinoside) (Campanile et al. 2022).

A study of hydroalcoholic extract of *R. graveolens* leaves (Iran) showed improvement in spatial memory and reduced latency in rats, with dose‐dependent effects. A similar effect was observed for rutin (10 mg/kg), confirming the contribution of this compound to cognitive effects (Asgharian et al. 2020).

The dichloromethane extract showed potent inhibition of monoamine oxidases (hMAO‐A—88.22%, hMAO‐B—89.98%), with low selectivity (SI = 1.02), indicating its potential ability to regulate monoaminergic pathways (Kozioł et al. 2020).

The data indicate that *R. graveolens* preparations exert neuroprotective effects through various mechanisms. They not only affect enzyme targets associated with neurodegeneration, but also contribute to reducing oxidative damage and inflammatory responses, as well as supporting neurotrophic mechanisms. This highlights the promise of further research into *R. graveolens* aimed at finding new preventive and therapeutic approaches to neurodegenerative diseases.

### 7.5 Anti‐Asthmatic Activity

The anti‐asthmatic potential of *R. graveolens* has been demonstrated in a rat model of ovalbumin‐induced allergic asthma. A methanolic extract and its nano‐sized cubosomal dispersion significantly subsided asthma severity scores and improved respiratory function by restoring the FEV1/FVC ratio to near‐normal values. The primary mechanism of action involves the modulation of the airway inflammatory microenvironment. Specifically, the treatment effectively suppresses the Th2‐driven immune response by decreasing the levels of pro‐inflammatory cytokines (IL‐4, IL‐7) and Ig‐E in bronchoalveolar lavage fluid, while simultaneously increasing IFN‐γ levels to restore the Th1/Th2 balance. Furthermore, the extract inhibits the expression of TGF‐β, which is crucial for preventing airway remodeling and subepithelial fibrosis. These anti‐inflammatory effects are complemented by potent antioxidant activity, as evidenced by increased SOD activity and reduced MDA levels in lung tissues (Alyami et al. 2024).

The antiasthmatic potential of *R. graveolens* was further confirmed by its direct bronchodilator effect on isolated rat tracheal rings. The aqueous‐alcoholic extract significantly reduced airway smooth muscle contractions induced by both carbachol and potassium chloride in a concentration‐dependent manner. At a concentration of 45 μg/mL, the extract achieved maximal relaxation values of 118% for carbachol and 109% for potassium chloride, with IC_50_ of 27.8 μg/mL and 35.5 μg/mL, respectively. These results indicate a relaxation activity comparable to that of the standard drug aminophylline. The primary mechanism of action was identified as non‐competitive antagonism. This effect probably involves blockade of ionic currents, preventing the influx of calcium required for muscle tension. These properties are explained by the presence of key secondary metabolites, including rutin, quercetin, and psoralen (Águila et al. 2015).

### 7.6 Antitumor Activity

*R. graveolens* is distinguished by a variety of secondary metabolites and proven cytotoxic and antiproliferative activity. Experimental studies have shown the effectiveness of its extracts in various in vitro and in vivo models (Table 6). In addition, there are a number of studies showing the feasibility of using *R. graveolens* for adjunctive therapy to reduce the side effects of certain drugs and improve well‐being in palliative care (Karp et al. 2016; Freyer et al. 2014).

**TABLE 6:** Antitumor activity of *R. graveolens* .

Cells	Extract	Result	References
Cutaneous melanoma	*R. graveolens* 9CH (ultra‐low homeopathic dilution)	*R. graveolens* 9CH modifies the physicochemical properties of cells, reducing rigidity and altering the lipid composition of membranes, accompanied by disruption of calcium flows and disorganization of the actin cytoskeleton. As a result, inhibition of B16F10 melanoma cell migration in vitro and reduction of metastasis in vivo (−50% of metastases in the lungs) were observed, which increased the survival rate of mice to 55% on day 23.	Fuselier et al. 2022
Breast cancer (reduction of side effects)	*R. graveolens* 5CH (in combination with *Rhus toxicodendron* 9CH)	The combination used significantly reduced the total joint pain score (−1.3 vs. +3.4 in the control group; *p* = 0.0001), the need for analgesics (1 patient vs. 9; *p* = 0.0076), the negative impact of pain on sleep (0% vs. 35%; *p* = 0.0083), and reduced the time to disappearance of morning stiffness (17.9 ± 20.1 min vs. 37.7 ± 23.0 min; *p* = 0.0173).	Karp et al. 2016
Human adult T‐cell leukemia/lymphoma	Methanolic (roots, leaves, and aerial parts)	The average antiproliferative activity of the extract from the roots (EC_50_: 10–100 μg/mL), leaves (10–100 μg/mL), and aerial parts (10–100 μg/mL) against MT‐1. High antiproliferative activity of roots (1–10 μg/mL), leaves (1–10 μg/mL), and aerial parts (10–100 μg/mL) against MT‐2.	Nakano et al. 2016
Melanoma cells (A375)	Ethanolic	The extract induces apoptosis in A375 cells (activation of BAX, caspase‐3, PARP, release of cytochrome c) and autophagy (increase in LC3‐II, Atg5, Atg7, Beclin‐1; decrease in mTOR, Bcl‐2), reduces pAkt; It is non‐toxic to PBMC and HaCaT; at a dose of 75 mg/kg, it reduces tumor burden in mice with DMBA‐induced skin cancer.	Ghosh et al. 2015
Glioblastoma multiforme (U87MG, C6 and U138)	Water	The extract induces apoptosis in glioblastoma cells (U87MG, C6, U138) and undifferentiated A1 neuronal cells by activating ERK1/2, AKT, and caspase‐3; viability decreased by 120% after 48 h and by 200% after 72 h. not toxic to differentiated A1 cells; rutin does not exhibit cytotoxicity.	Gentile et al. 2015
Glioblastoma (U87MG, U251MG, GSCs)	Water	Inhibits survival, migration, and invasion; suppresses vasculogenic mimicry at sub‐lethal doses. Effect not reproduced by rutin, suggesting synergism of multiple components.	Camerino et al. 2024
Breast cancer (MCF7)	Methanolic	The extract induces morphological signs of apoptosis and reduces the viability of MCF‐7 cells in a dose‐dependent manner; IC_50_ = 160 μg/mL.	Pushpa et al. 2015
Breast cancer (MCF‐7), Cervical cancer (HeLa), Leukemia (Jurkat), Bladder cancer (T24), Colon cancer (HT‐29)	Essential oil	High potency after elicitation (IC50) HeLa (4.3 μg/mL), MCF‐7 (8.11 μg/mL), Jurkat (17.05 μg/mL), T24 (82.2 μg/mL), HT29 (70.53 μg/mL). Induces Caspase‐3/7‐mediated apoptosis. Selective towards cancer cells over normal HEK‐293. Bioactivity driven by ketones (2‐undecanone and 2‐nonanone).	Mahmoud et al. 2020
Cervical cancer (HeLa), Liver cancer (HepG2), Melanoma (B16‐F1)	Essential oil	Dose‐dependent cytotoxicity (IC_50_) HepG2 (554.47 μg/mL), B16‐F1 (752.90 μg/mL), HeLa (830.90 μg/mL). Inhibits AMPA receptor current 3‐fold and increases deactivation rate 1.5‐fold.	Qneibi et al. 2025
Lung cancer (NSCLC, EGFR_T790M)	Ethyl acetate (leaves)	Potent inhibitor of drug‐resistant mutant; higher binding affinity to mutant kinase (Kd = 0.67 μg); inhibits EGFR phosphorylation (Tyr1068/1173); induces G1/G0 phase arrest and apoptosis. Bioactive molecules: Balticol F, Komunesin A, Tylanstatin A.	Kumar et al. 2025

Abbreviations: A1, Neuronal cells; A375, Human melanoma cell line; AKT, Protein Kinase B; AMPA, α‐amino‐3‐hydroxy‐5‐methyl‐4‐isoxazolepropionic acid; Atg5/Atg7, Autophagy‐related proteins 5 and 7; B16‐F1/B16F10, Mouse melanoma cell lines; BAX, Bcl‐2‐associated X protein; Bcl‐2, B‐cell lymphoma 2; C6, Glioblastoma cell line; CH, Centesimal Hahnemannian dilution; DMBA, 7,12‐Dimethylbenzanthracene; EC_50_, Half maximal effective concentration; EGFR, Epidermal Growth Factor Receptor; ERK1/2, Extracellular signal‐regulated kinases 1 and 2; G0/G1, Phases of the cell cycle; GSCs, Glioma Stem Cells; HaCaT, Human immortalized keratinocyte cell line; HEK‐293, Human Embryonic Kidney cells; HeLa, Human cervical cancer cell line; HepG2, Human liver cancer cell line; HT‐29, Human colon cancer cell line; IC_50_, Half maximal inhibitory concentration; Jurkat, Human T lymphocyte cell line (leukemia); Kd, Dissociation constant; LC3‐II, Microtubule‐associated protein 1 light chain 3 (autophagy marker); MCF‐7, Human breast cancer cell line; MT‐1/MT‐2, Human T‐cell leukemia cell lines; mTOR, Mammalian target of rapamycin; NSCLC, Non‐small‐cell lung cancer; pAkt, Phosphorylated (active) form of Protein Kinase B; PARP, Poly (ADP‐ribose) polymerase; PBMC, Peripheral Blood Mononuclear Cells; T24, Human bladder cancer cell line; U87MG/U138/U251MG, Human glioblastoma cell lines.

Studies on breast cancer (MCF‐7) showed that the methanol extract of the aerial parts reduced cell viability in a dose‐dependent manner (IC_50_ 160 μg/mL), causing morphological signs of apoptosis (cytoplasmic vacuolization, fragmentation, cell detachment, membrane deformation) (Pushpa et al. 2015).

The aqueous extract of the aerial parts of *R. graveolens* has been found to have a cytotoxic effect on glioblastoma cells (U87MG, U138, C6). The extract induced caspase‐3‐dependent apoptosis through the activation of ERK1/2 and AKT. The drug selectively acted on proliferating glioma cells without damaging differentiated neurons. Rutin, the main component of the extract, did not reproduce the effects, indicating other active compounds (Gentile et al. 2015). Another study showed that an aqueous extract of *R. graveolens* significantly inhibited not only the survival but also the migration and invasion of human glioblastoma cells. Importantly, sublethal doses impaired vasculogenic mimicry, the ability of aggressive tumor cells to form vessel‐like structures to provide a blood supply. This effect was observed in both established cell lines and glioblastoma cancer stem cells obtained from patients, which are usually resistant to standard chemotherapy. Notably, rutin alone did not reproduce these antimetastatic effects, suggesting that the efficacy of the entire extract arises from the synergistic interaction of multiple bioactive compounds (Camerino et al. 2024).

Rutin extract also showed activity against melanoma (A37). It induced mitochondrial‐dependent apoptosis (BAX, caspase‐3, PARP, cytochrome c) and autophagy (Atg5, Atg7, Beclin‐1, LC3‐II, mTOR, and Bcl‐2 reduction). It showed selective cytotoxicity to tumor cells without harming PBMC and HaCaT. Oral administration to mice (75 mg/kg) reduced tumor burden, decreased pAkt, and enhanced antioxidant protection without toxicity (Ghosh et al. 2015).

With regard to leukemia/lymphoma (MT‐1, MT‐2), methanol extracts of leaves, aerial parts, and roots demonstrated a pronounced antiproliferative effect, with dihydrofuranocoumarin chalepin proving to be the most active (Nakano et al. 2016).

Recent studies have identified ethyl acetate extract of *R. graveolens* leaves as a specific and potent inhibitor against drug‐resistant non‐small cell lung cancer harboring the EGFR_T790M mutation. The extract exhibits higher binding affinity to the mutant EGFR kinase compared to the wild‐type protein. Mechanistically, the extract inhibits tumor progression by inhibiting EGFR phosphorylation at Tyr1068 and Tyr1173 residues, leading to dose‐dependent cell cycle arrest in the G1/G0 phase and induction of apoptosis. Targeted mass spectrometry has identified several key bioactive molecules responsible for this effect, including Balticol F, Communesin A, and Thailanstatin A (Kumar et al. 2025). Experimental studies also confirm the efficacy of its extracts on a wide range of cancer cell lines, including breast adenocarcinoma (MCF‐7), cervical cancer (HeLa), bladder cancer (T24), colon cancer (HT‐29), and T‐cell leukemia (Jurkat). The primary mechanism of cytotoxic action is the intrinsic apoptotic pathway triggered by caspase‐3/7 activation, cytochrome c release, and DNA fragmentation, leading to a significant accumulation of necrotic and apoptotic cells. The biological activity in the first place is explained by the high concentration of ketones, including 2‐undecanone and 2‐nonanonate (Mahmoud et al. 2020). Another study showed that the essential oil, characterized by high content of 2‐undecanone and 2‐nonanone, inhibited cervical adenocarcinoma (HeLa), hepatocellular carcinoma (HepG2) and mouse melanoma (B16‐F1) cells. Quantitative analysis showed IC50 values of 830.90 μg/mL for HeLa, 554.47 μg/mL for HepG2 and 752.90 μg/mL for B16‐F1 cells. Although the summer fraction showed significant activity in these specific models, it highlights the broader pharmacological potential of the plant in affecting a variety of malignant phenotypes (Qneibi et al. 2025).

Several studies of homeopathic preparations based on *R. graveolens* are also known. For example, *R. graveolens* 9CH inhibited the migration of B16F10 melanoma cells by altering the actin cytoskeleton, membrane stiffness, and calcium flux. In a metastasis model, the number of nodes in the lungs decreased by 50%, and mouse survival increased to 55% on days 22–23 (Fuselier et al. 2022).

In a clinical study in patients with terminal stages of cancer, oral administration of *R. graveolens* 9CH (2–3 months) improved quality of life but did not affect tumor progression, indicating palliative potential (Freyer et al. 2014).

In women with early hormone‐dependent breast cancer, *R. graveolens* 5CH in combination with *Rhus toxicodendron* 9CH reduced joint pain and stiffness associated with aromatase inhibitor therapy, improving quality of life (Karp et al. 2016).

Thus, *R. graveolens* exhibits multifaceted antitumor activity and it induces apoptosis and autophagy, inhibits tumor cell migration, reduces tumor burden in vivo, and also exhibits palliative potential in clinical settings. The activity is due not only to rutin, but also to other components of the extract (chalepin), indicating a synergistic mechanism of action.

### 7.7 Antivitiligo Activity

In a study by Chinese researchers, methanol extract of *R. graveolens* , which was subsequently divided into different fractions, showed promising activity in an in vitro vitiligo model, stimulating melanogenesis and protecting melanocytes from oxidative damage. In B16F10 cells, the extract was found to significantly increase melanin synthesis, tyrosinase, TRP‐1, and TRP‐2 activity, as well as the expression of the main regulator of melanogenesis, MITF. In addition, an increase in phosphorylated CREB levels was observed, confirming the activation of the corresponding signaling pathway (Ainiwaer et al. 2023).

### 7.8 Activity of Individual Compounds

Individual compounds isolated from the plant demonstrate a wide range of biological effects, confirming their role as key active ingredients. The study of individual components allows for a more detailed characterisation of the mechanisms of action, determination of pharmacokinetic parameters, identification of possible targets, and assessment of therapeutic potential. This section summarizes data on the most studied substances of *R. graveolens* , such as cocusaginine, rutin, graveolin, rutamarin, skimmianine, and others, which exhibit antifibrotic, hepatoprotective, sensory, antitumor, and other activities (Table 7).

**TABLE 7:** Activity of individual compounds of *R. graveolens* .

Compound	Result	References
Alkaloids		
Kokusaginine	*Antifibrotic activity*: cocusaginine reduced TGF‐β‐induced expression of fibronectin, collagen I, and α‐SMA in NRK‐49F and HK‐2 cells; improved renal function and reduced fibrosis in mice with renal fibrosis. The mechanism of action is associated with inhibition of the PI3K/AKT signaling pathway.	Wang et al. 2024
Kokusaginine	*Cytoprotective activity*: melanin activation 185.3%; at 50 μM—melanin 170.1%; protection of PIG3V melanocytes; reduction of ROS and IL‐6 levels.	Ainiwaer et al. 2023
Kokusaginine	*Pharmacokinetic activity*: rapid absorption after oral administration (28 mg/kg), absolute bioavailability = 71.13% ± 12.75%, half‐lives *t*_1/2_ in liver microsomes: males—7.76 min, females—69.73 min; CLint*ₜ* (liver, males) = 89.30 μL/min/mg; CLint (liver, females) = 9.94 μL/min/mg; highest accumulation—liver, kidneys; detected in the brain.	Shang et al. 2024
Graveoline	*Hepatoprotective and anti‐inflammatory activity*: reduced ALT, AST, and TNF‐α levels, increased IL‐4 and IL‐10 in models of acute liver injury (LPS, D‐GalN/LPS). The mechanism of action is associated with inhibition of the JAK1/STAT3 signaling pathway.	He et al. 2024
Graveoline	*Cytotoxic activity against skin cancer cells*: exhibited dose‐dependent cytotoxicity against A375 melanoma cells, inducing both apoptosis and autophagic death mediated by the generation of reactive oxygen species. It reduced the expression of mTOR and Bcl‐2 and increased the levels of LC3‐II, Atg5, Atg7, and Beclin‐1 without causing significant death of normal cells.	Ghosh et al. 2013
Skimmianine	*Activity associated with the activation of bitter taste receptors*: selective agonist of the bitter receptor TAS2R14 (IC_50_ ≥ 15.8 μM).	Mancuso et al. 2015
Gravacridonediol	*Antibacterial activity*: has high affinity against InhA *Mycobacterium tuberculosis* (Binding Energy = −10.49 kcal/mol; Ki (Inhibition Constant) 600.24 nM); binds to the active site of InhA, forming hydrogen bonds.	Singh et al. 2022
Arborinine	*Cytotoxic activity against skin cancer cells*: selective cytotoxicity against MCF‐7 breast cancer cells IC_50_ = 50 μg/mL at 24 h, 25 μg/mL at 48 h); non‐toxic to normal HEK293 cells (> 150 μg/mL). Activation of the intrinsic apoptotic pathway (increased BAX, P53, PARP, caspases 3/8/9; decreased Bcl‐2) and induction of oxidative stress (ROS increased 1.3‐fold).	Zangouri et al. 2025
Coumarins		
Bergapten	*Selective receptor activity*: potent agonist of bitter receptor TAS2R10 (EC_50_ 2.8 μM)	Mancuso et al. 2015
Xanthotoxin	*Selective receptor activity*: agonist of TAS2R10 (EC_50_ ≥ 20.6 μM), TAS2R14 (EC_50_ ≥ 10.8 μM), TAS2R49 (EC_50_ 12 μM) and weak activator of TRPA1 (EC_50_ ≥ 19.7 μM)	Mancuso et al. 2015
Isopimpinellin	*Selective receptor activity*: weak agonist of TAS2R10 (EC_50_ = 12 μM) and TAS2R14 (EC_50_ 11.1 μM), activator of TRPA1 (EC_50_ ≥ 22.8 μM).	Mancuso et al. 2015
8‐methoxychromen‐2‐one	*Anti‐inflammatory and anti‐arthritic activity*: dose‐dependent inhibition of NO, iNOS, IL‐1β, NF‐κB; 75% and 84% reduction in arthritic index (2 and 20 mg/kg), reduction in joint swelling and destruction, reduction in TNF‐α, IL‐1β, IL‐6.	Sahu et al. 2015
Rutamarin	*Ion channel modulating activity*: activated receptor TRPM5 (EC_50_ ≥ 26.7 μM) and TRPV1, exhibited strong antagonistic activity on TRPM8 (IC_50_ 124 μM).	Mancuso et al. 2015
Rutamarin	*Selective MAO‐B inhibitory activity*: isolated rutamarin exhibited inhibition of the hMAO‐B enzyme (95.26%) with significantly lower activity against hMAO‐A (25.15%), with a selectivity index SI = 3.788.	Kozioł et al. 2020
Rutamarin	*Antiviral activity*: (−)‐rutamarin inhibits Epstein–Barr Virus replication (IC_50_ 13.8 μM), similar to (+)‐rutamarin (IC_50_ = 7.0 μM); SI 4.0	Lin et al. 2017
Flavonoids		
Rutin	*Antifibrotic and nephroprotective activity*: reduced IgA and C3 deposition in the glomeruli, decreased serum creatinine, urea nitrogen, and albuminuria, and inhibited IL‐6, TGF‐β, and α‐SMA expression by inhibiting the TGF‐β/SMAD pathway. This led to a reduction in inflammation and fibrosis.	Jash et al. 2024

Abbreviations: α‐SMA, Alpha‐smooth muscle actin; A375, Human melanoma cell line; ALT, Alanine aminotransferase; AST, Aspartate aminotransferase; Atg5/Atg7, Autophagy‐related proteins 5 and 7; BAX—Bcl‐2‐associated X protein; Beclin‐1, Autophagy‐related protein Beclin‐1; Bcl‐2, B‐cell lymphoma 2; C3, Complement component 3; CLint, Intrinsic clearance; D‐GalN/LPS, D‐galactosamine/Lipopolysaccharide; EC_50_, Half maximal effective concentration; hMAO‐A/B, Human Monoamine Oxidase A and B; HK‐2, Human kidney proximal tubular cell line; IC_50_, Half maximal inhibitory concentration; IgA, Immunoglobulin A; IL‐1β/IL‐4/IL‐6/IL‐10, Interleukins; InhA, Enoyl‐ACP reductase (target for tuberculosis treatment); iNOS, Inducible nitric oxide synthase; JAK1/STAT3, Janus kinase 1/Signal transducer and activator of transcription 3 signaling pathway; Ki, Inhibition constant; LC3‐II, Microtubule‐associated protein 1 light chain 3; LPS, Lipopolysaccharide; mTOR, Mammalian target of rapamycin; NF‐κB, Nuclear factor kappa‐light‐chain‐enhancer of activated B cells; NO, Nitric oxide; NRK‐49F, Normal rat kidney interstitial fibroblast cell line; P53, Tumor protein p53; PARP, Poly (ADP‐ribose) polymerase; PI3K/AKT, Phosphoinositide 3‐kinase/Protein Kinase B signaling pathway; PIG3V, Human vitiligo melanocyte cell line; ROS, Reactive oxygen species; SI, Selectivity index; SMAD, Mothers Against Decapentaplegic Homolog; *t*_1/2_, Half‐life; TAS2R10/14/49, Bitter taste receptors; TGF‐β, Transforming growth factor beta; TNF‐α, Tumor necrosis factor alpha; TRPA1/TRPM5/TRPM8/TRPV1, Transient receptor potential ion channels.

#### 7.8.1 Antifibrotic Activity

Coccusagin, isolated from *R. graveolens* , demonstrated a pronounced anti‐fibrotic effect on the kidneys in in vitro and in vivo models. In NRK‐49F and HK‐2 cells, it reduced TGF‐β‐induced expression of fibronectin, collagen I, and α‐SMA, inhibiting fibroblast activation and epithelial‐mesenchymal transition. In mice with renal fibrosis, coccusagin improved renal function, reduced histological damage and the degree of fibrosis, with a mechanism of action related to PI3K/AKT inhibition (Wang et al. 2024).

Pharmacokinetic studies have shown that cocusaginine is rapidly absorbed after oral administration (28 mg/kg), has high bioavailability (71.13%), and is evenly distributed in tissues, with the highest accumulation in the liver and kidneys. The compound is able to cross the blood–brain barrier and exhibits sex‐specific differences in metabolism, but requires monitoring for liver effects at high doses (Shang et al. 2024).

Rutin in a rat model of IgA nephropathy reduced IgA and C3 deposition in the glomeruli, lowered creatinine, urea nitrogen, and albuminuria levels, and reduced red blood cells in the urine. Rutin treatment suppressed inflammatory and fibrotic markers (IL‐6, TGF‐β, α‐SMA) by inhibiting the TGF‐β/SMAD pathway, which prevented epithelial‐mesenchymal transition (Jash et al. 2024).

#### 7.8.2 Hepatoprotective Activity

Graveolin demonstrated hepatoprotective activity in models of acute liver injury in vitro and in vivo. The compound reduced ALT and AST levels, suppressed the proinflammatory cytokine TNF‐α, and activated the anti‐inflammatory cytokines IL‐4 and IL‐10. The mechanism of action is associated with the inhibition of the JAK1/STAT3 signaling pathway, as confirmed by biochemical studies and the KEGG network. However, a dose of 100 mg/kg was excessive and caused signs of hepatotoxicity, requiring further evaluation of the safe range (He et al. 2024).

#### 7.8.3 Sensory and Nociceptive Activity

Compounds from *R. graveolens* exhibited activity through interaction with TAS2R bitter taste receptors and TRP ion channels. Rutamarin acted as an agonist of TRPM5 and TRPV1 and an antagonist of TRPM8, potentially reducing cold nociception. Skimmianin was a selective agonist of TAS2R14, bergapten was an agonist of TAS2R10, and xanthotoxin activated TAS2R10, TAS2R14, TAS2R49, and TRPA1. Isopimpinellin activated TAS2R10, TAS2R14, and TRPA1. Essential oil from the leaves showed strong and selective activity toward TAS2R14, indicating the role of these compounds in sensory, taste, and nociceptive responses (Mancuso et al. 2015).

#### 7.8.4 Antiarthritic Activity

8‐Methoxychromen‐2‐one (MCO), isolated from the hydromethanol extract of the aerial parts of *R. graveolens* , showed significant anti‐arthritic activity. In vitro, it dose‐dependently inhibited NO, iNOS, IL‐1β, and NF‐κB in LPS‐stimulated J774 macrophages. In a collagen‐induced arthritis model in rats, MCO (2 and 20 mg/kg) reduced the arthritis index by 75%–84%, decreased paw swelling, joint destruction, and TNF‐α, IL‐1β, and IL‐6 levels, and improved behavioral parameters. Histological and radiological studies confirmed a reduction in inflammation and joint damage without signs of toxicity (Sahu et al. 2015).

#### 7.8.5 Antimycobacterial Activity

Gravaquidione, the main glycosylated alkaloid of *R. graveolens* , is a potent inhibitor of the *Mycobacterium tuberculosis* InhA enzyme, which is key in the biosynthesis of mycolic acids. The compound showed high binding affinity (E.Z. −10.49 kcal/mol; Ki = 600.24 nM) through hydrogen and π–π bonds with amino acid residues of the active site (Ile194, Pro193, Val65, Gly14), exceeding the efficacy of the known inhibitor triclosan (Singh et al. 2022).

#### 7.8.6 Antiviral Activity

(−) and (+)‐Rutamarin from *R. graveolens* showed activity against Epstein–Barr virus (EBV) DNA replication with IC_50_ values of 13.8 and 7.0 μM, respectively (Lin et al. 2017).

#### 7.8.7 Monoamine Oxidase Inhibition

Rutamarin was isolated from the dichloromethane extract of *R. graveolens* and showed high selectivity for inhibiting hMAO‐B (95.26%) with low activity against hMAO‐A (25.15%), with a selectivity index SI = 3.79, almost four times higher than that of the crude extract (Kozioł et al. 2020).

#### 7.8.8 Antitumor Activity

Graveolin from the ethanol extract of *R. graveolens* showed cytotoxicity against A375 melanoma cells, inducing apoptosis and autophagic death. MTT analysis showed a dose‐dependent decrease in viability, while normal mononuclear cells remained almost unaffected. Graveolin reduced the expression of mTOR and Bcl‐2, increased LC3‐II, Atg5, Atg7, and Beclin‐1, and caused the generation of reactive oxygen species that mediate cell death. Apoptosis inhibition did not block autophagy, indicating the independence of these pathways (Ghosh et al. 2013).

Arborinine has also been shown to exhibit selective cytotoxicity against MCF‐7 cells with IC_50_ values of 50 μg/mL (24 years) and 25 μg/mL (48 years), while significantly higher concentrations (> 150 μg/mL) are required to affect normal kidney cells (HEK293). The mechanism of action involves the activation of the intrinsic apoptotic pathway, which promotes the increase in BAX, P53, PARP and caspases 3, 8 and 9, as well as the decrease in Bcl‐2 levels. In addition, treatment with arborinine induces oxidative stress, increasing the level of ROS by 1.3 times and decreasing the level of glutathione (Zangouri et al. 2025).

#### 7.8.9 Antivitiligo Activity

Cocusagenin, an alkaloid from *R. graveolens* , has demonstrated potential activity in the prevention and treatment of vitiligo by affecting cellular pigmentation mechanisms. It reduced ROS, IL‐6, and ER stress protein expression in human PIG3V melanocytes, protecting cells from oxidative damage (full text needs clarification in the source) (Ainiwaer et al. 2023).

The studied extracts and individual compounds of *R. graveolens* demonstrate a wide range of pharmacological activities: anti‐inflammatory, antioxidant, antimicrobial, antitumor, neuroprotective, hepatoprotective, antiviral, and anti‐arthritic. Both alcohol and water extracts were found to be the most active, confirming the importance of the complex effects of secondary metabolites, including flavonoids, coumarins, and alkaloids. The extracts exhibit different specificities depending on the extraction method and solvent used, allowing the selection of optimal methods for therapeutic applications. The main bioactive components are alkaloids (graveolin, cocusaginine, gravaquidione), coumarins (rutamarin, bergapten, xanthotoxin), flavonoids, and volatile essential oils. The mechanisms of action are often associated with the regulation of key signaling pathways (TGF‐β/SMAD, PI3K/AKT, NF‐κB, JAK/STAT), modulation of cytokine profiles, antioxidant and anti‐inflammatory activity. Further research should focus on standardizing extracts, studying the mechanisms of action of active components, and developing safe and bioavailable dosage forms.

## 8 Toxic Properties of *R. graveolens*

Despite the fact that *R. graveolens* is actively used in traditional medicine and cooking, it can also cause phytophotodermatitis. This form of toxicity occurs as a result of skin contact with the plant's sap. The sap contains furanocoumarins, which can cause burns when exposed to ultraviolet radiation (Grosu Dumitrescu et al. 2024). There are various phytotoxic compounds, such as psoralen, bergapten, isopimpinellin, isoimperatorin, chalepensin, marmesin, isorutarin, which are known to be phototoxic with a high level of evidence (Foresto et al. 2021), furanocoumarins 5‐ and 8‐methoxypsoralen (Gawkrodger and Savin 1983; Heskel et al. 1983), and graveolin (Eickhorst et al. 2007).

In general, phytophotodermatitis is a non‐allergic inflammatory reaction; therefore, it does not require prior sensitization of the body but is phototoxic, manifested by redness, burning, itching, swelling, blistering, their fusion, erosion, ulcers, and subsequently hyperpigmentation, hyperkeratosis (Gawkrodger and Savin 1983; Eickhorst et al. 2007; Heskel et al. 1983; Pesqué et al. 2024). One of the known mechanisms of toxicity is the activation of furocoumarins by ultraviolet light, which leads to damage to the DNA of skin cells by inhibiting mitosis and inflammation (Eickhorst et al. 2007). Cases of phytophotodermatitis have been reported in the literature in connection with the use of the plant in local medicine (Córdoba et al. 2017; Gawkrodger and Savin 1983) or accidental contact in nature (Heskel et al. 1983; Eickhorst et al. 2007). Treatment includes topical corticosteroids, antihistamines, soothing baths to relieve itching, and avoidance of further ultraviolet exposure (Eickhorst et al. 2007).

The presence of furocoumarins and other phototoxic compounds in the leaves, stems, sap, and fruits of the plant highlights the importance of recognizing these causes and taking precautions to minimize the risk of skin reactions (Grosu Dumitrescu et al. 2024). It is reported that *R. graveolens* essential oil also contains psoralens, which cause phototoxicity (Heskel et al. 1983).

Equally important is the fact that the toxicity of *R. graveolens* is not limited to local reactions. According to recent studies, the plant contains furocoumarins, coumarins, acridones, and other active compounds, among which chalepensin, known as a potent and dangerous anti‐fertility agent, attracts attention. It has been proven that extracts from various parts of the plant can cause implantation disorders, fetal death, organ toxicity, and even renal failure. The use of *R. graveolens* as an emmenagogue and abortifacient has a long tradition and is confirmed in ethnomedical sources (Ciganda and Laborde 2003; Madari and Jacobs 2004; Tobyn et al. 2011). Its phytotoxic potential has been demonstrated in *Allium cepa* models, where extract significantly inhibited root growth, indicating a narrow therapeutic index where even a slight increase in dosage can lead to toxic effects (Abebe 2021). Other data show that exposure of *Caenorhabditis Elegans* nematodes to *R. graveolens* extract in the early stages caused a slowdown in population growth and irreversible reproductive dysfunction. The results provide some evidence of the plant's toxicity in certain dosages and may serve as a potential warning about the dangers to reproductive function and pregnant women (Abebe 2023).

The results of a study in mice infected with *Vampirolepis nana* showed that hydroalcoholic extracts of *R. graveolens* leaves and seeds exhibit only weak antiparasitic activity, but are accompanied by pronounced toxic effects. High doses caused an increase in liver enzyme activity (AST, ALT), mild to moderate hepatotoxicity, a decrease in the albumin/globulin ratio, normochromic‐normocytic anemia, neutropenia, and behavioral disorders. In the context of unstandardized self‐medication, these data confirm that the plant is a dangerous remedy, since its toxicity significantly exceeds its potential therapeutic efficacy in crude preparations (Freire et al. 2010). Another experiment with ethanol and water extracts of *R. graveolens* seeds in rats demonstrates weight loss, negative changes in blood serum parameters, decreased globulins and urea, significant liver and kidney damage, and increased mortality when administered orally at a dose of 200 mg/kg/day for 4 weeks. However, the use of the extract at a dose of 50 mg/kg/day is considered safe for use, as there were no changes in any of the measured parameters (Adam et al. 2014).

There is also a clinical case of systemic poisoning with *R. graveolens* decoction resulting in bradycardia, acute renal failure with hyperkalemia, and coagulopathy, requiring hemodialysis (Seak and Lin 2007).

In conclusion, the apparent contradiction in the literature, where the plant is called both a “dangerous remedy” and a “promising pharmacological object”, is resolved by distinguishing the context of use. Although unstandardized crude preparations used in local medicine pose a risk due to the uncontrolled presence of phototoxic furanocoumarins and abortifacient alkaloids, *R. graveolens* remains a promising pharmacological object when considering isolated bioactive fractions or purified extracts and advanced delivery systems. Also, building on this distinction, future research must prioritize the systematic isolation and purification of individual metabolites to fully delineate their specific pharmacological roles and safety profiles. These modern scientific approaches allow for the selective preservation of therapeutic effects while excluding components responsible for systemic and phototoxic reactions. Through careful dosage control and strict standardization of the raw material, *R. graveolens* can be transformed from a high‐risk ethnomedicinal product into a highly effective medicinal product. Further research should be aimed at clarifying its safety, optimizing methods of application, and revealing the potential of bioactive components for medicine and pharmacy.

## 9 Conclusions and Future Perspectives

*Ruta graveolens* L. is a promising phytomedicine object due to its extremely rich chemical composition, including more than 160 identified secondary metabolites, as well as a wide range of traditionally described and experimentally confirmed pharmacological effects, including antimicrobial, antifungal, antiviral, antitumor, neuroprotective, antioxidant, etc. At the same time, despite a significant number of preclinical studies, the current evidence base for *R. graveolens* remains fragmented and uneven. Most of the available pharmacological data are based on in vitro and in vivo studies, while clinical data are limited and mainly represented by pilot or observational studies. Individual clinical reports concern the reduction of joint pain in patients receiving aromatase inhibitors or the use of homeopathic doses in adjuvant cancer therapy. However, to date, there are no randomized controlled clinical trials aimed at assessing key therapeutic effects for humans which significantly limits its comparability with other medicinal plants. This gap is critical, as clinical evidence is currently restricted to observations in palliative care or the use of ultra‐low homeopathic dilutions. This limitation is due to insufficient studies of both the chemical composition of the raw materials and biotechnological methods and, of course, insufficient preclinical studies of *R. graveolens* .

Certain areas of pharmacological research, in particular antiviral activity, are limited to in vitro results without in vivo confirmation, which does not allow making reasonable conclusions about clinical potential. In addition, comparative studies with other species of the genus *Ruta* (e.g., *R. chalepensis* ) or with other members of the Rutaceae family are not sufficiently presented, which could contribute to a better understanding of the pharmacological features of the genus.

An important aspect that requires further study is the safety of the use of this plant. The analysed sources indicate potential risks of toxicity, in particular the presence of described cases of poisoning, phototoxic reactions and reproductive effects. While the phototoxicity of furanocoumarins is well‐documented, there is a notable lack of systematic long‐term studies aimed at assessing chronic toxicity and drug interactions. Furthermore, more detailed investigations into gender‐specific differences in sensitivity to *R. graveolens* compounds and their precise impact on reproductive functions are required to establish a comprehensive safety profile.

Phytochemical studies have identified a wide range of bioactive compounds, but the factors determining the variability of chemical composition remain poorly understood. In particular, there are no systematic data on the influence of the collection season, soil type, climatic conditions, and cultivation methods on the quantitative and qualitative profile of secondary metabolites. Even for rutin, which is often considered a marker compound, significant variability in content is observed, while universal standards for extracts, similar to pharmacopoeial requirements for other medicinal plants, have not yet been developed.

*R. graveolens* is an adaptable plant that is cultivated in many countries around the world, but data on sustainable cultivation, impacts on biodiversity, accumulation of pollutants, and environmental risks remain limited. Ethnobotanical information is largely historical, while current systematic surveys and regional studies of *R. graveolens* use are incomplete.

Future research on *R. graveolens* should move from basic experimental work to applied research with a clear focus on safety and efficacy. A promising approach is to initiate randomized controlled clinical trials for the most substantiated areas, including neuroprotection (e.g., in Alzheimer's disease or ischemic injuries, taking into account inhibition of AChE and BChE), vitiligo therapy (through effects on melanogenesis), and others. Such studies should be based on the use of standardized extracts with a defined content of marker compounds, with mandatory placebo control and monitoring of side effects, especially in vulnerable populations.

Particular attention should be paid to expanding toxicological studies, including long‐term experiments in animals and humans, as well as developing protocols for the safe use of rue‐based drugs. An important task is the development of methods for standardizing plant raw materials and extracts in accordance with pharmacopoeial requirements, as well as comparative analysis of samples from different regions and countries.

A promising direction is also an in‐depth study of the molecular mechanisms of action and synergy of individual components of the chemical composition, the creation of phytopreparations with controlled dosage and the potential study of combinations with other plants. The variability of the chemical composition depending on the origin of the raw materials makes it necessary to conduct local studies, and during clinical trials it is advisable to take into account the cultural traditions of using the plant.

Another promising research frontier lies in the isolation of individual bioactive compounds from *R. graveolens* and the detailed investigation of their specific pharmacological activities. While the complex effect of crude extracts is valuable, characterizing the mechanisms of action, pharmacokinetic parameters, and therapeutic targets of isolated substances (such as kokusaginine, graveoline, and rutamarin) is essential for developing targeted phytopharmaceuticals. Such studies would allow for the identification of key active ingredients responsible for particular effects and facilitate the creation of high‐efficacy drugs with precisely controlled dosages.

Given the growing interest in natural medicines, *R. graveolens* can be considered as a potential raw material base for the development of new drugs. Additional prospects are opening up in the field of biotechnology, in particular genetic and breeding approaches aimed at reducing toxicity. From an economic point of view, *R. graveolens* may have export potential for regions with suitable growing conditions. At the same time, the integration of *R. graveolens* into official pharmacopoeias is possible only if the existing scientific gaps are filled and convincing evidence of safety and efficacy is provided.

## Author Contributions

**Liudas Ivanauskas:** investigation, data curation, writing – review and editing. **Victoriya Georgiyants:** writing – review and editing, project administration. **Tetiana Serhiienko:** conceptualization, writing – original draft, visualization, formal analysis, investigation. **Olha Mykhailenko:** supervision, writing – review and editing, conceptualization, methodology.

## Funding

The authors have nothing to report.

## Conflicts of Interest

The authors declare no conflicts of interest.

## Data Availability

Data sharing is not applicable to this article as no new data were generated or analysed in this study.
